# Bayesian meta‐analytical methods to incorporate multiple surrogate endpoints in drug development process

**DOI:** 10.1002/sim.6776

**Published:** 2015-11-03

**Authors:** Sylwia Bujkiewicz, John R. Thompson, Richard D. Riley, Keith R. Abrams

**Affiliations:** ^1^ Biostatistics Research Group, Department of Health SciencesUniversity of LeicesterUniversity RoadLeicesterLE1 7RHU.K.; ^2^ Genetic Epidemiology Group, Department of Health SciencesUniversity of LeicesterUniversity RoadLeicesterLE1 7RHU.K.; ^3^ Research Institute of Primary Care and Health SciencesKeele UniversityStaffordshireST5 5BGU.K.

**Keywords:** Bayesian analysis, multivariate meta‐analysis, multiple outcomes, surrogate endpoints, multiple sclerosis

## Abstract

A number of meta‐analytical methods have been proposed that aim to evaluate surrogate endpoints. Bivariate meta‐analytical methods can be used to predict the treatment effect for the final outcome from the treatment effect estimate measured on the surrogate endpoint while taking into account the uncertainty around the effect estimate for the surrogate endpoint. In this paper, extensions to multivariate models are developed aiming to include multiple surrogate endpoints with the potential benefit of reducing the uncertainty when making predictions. In this Bayesian multivariate meta‐analytic framework, the between‐study variability is modelled in a formulation of a product of normal univariate distributions. This formulation is particularly convenient for including multiple surrogate endpoints and flexible for modelling the outcomes which can be surrogate endpoints to the final outcome and potentially to one another. Two models are proposed, first, using an unstructured between‐study covariance matrix by assuming the treatment effects on all outcomes are correlated and second, using a structured between‐study covariance matrix by assuming treatment effects on some of the outcomes are conditionally independent. While the two models are developed for the summary data on a study level, the individual‐level association is taken into account by the use of the Prentice's criteria (obtained from individual patient data) to inform the within study correlations in the models. The modelling techniques are investigated using an example in relapsing remitting multiple sclerosis where the disability worsening is the final outcome, while relapse rate and MRI lesions are potential surrogates to the disability progression. © 2015 The Authors. *Statistics in Medicine* Published by John Wiley & Sons Ltd.

## 1 Introduction

Surrogate endpoints are increasingly being investigated as candidate endpoints in randomised controlled trials where measuring a primary outcome of interest may be too costly, too difficult to measure or require long follow‐up time. Prior to the use of surrogate endpoint for trial design or decision‐making for regulatory or reimbursement purposes, such endpoints need to be validated. The validation takes place on three levels: by establishing a biological plausibility of the association between outcomes, assessing association between outcomes at the individual level and validating the surrogate endpoints at the study level to assess them as predictors of clinical benefit measured by the final outcome 1. It has been established that methods based on a single clinical trial are not sufficient and surrogate endpoints have to be validated based on a number of clinical trials in a meta‐analytic framework 1, 2.

A number of meta‐analytical methods have been proposed that aim to evaluate treatment effects on surrogate endpoints as predictors of the effect on a target outcome. Methods by Buyse *et al*. 3 were developed to model surrogate endpoints at the arm level by extending the ideas developed by Prentice 4 to a meta‐analytic framework, while those by Daniels and Hughes 2 are focussed on modelling the relationship between relative treatment effects on outcomes. The former are developed in a frequentist approach while the latter in a Bayesian framework. Various extensions to meta‐analytic approaches to evaluating surrogate endpoints have been developed, for example, for the time‐to‐event data by Burzykowki *et al*. 5 extended to a Bayesian framework by Renfro *et al*. 6.

Most methods developed to date are designed to evaluate single surrogate endpoints. In the summary of a National Institutes of Health Workshop on the use of surrogate endpoints, Gruttola *et al*. 7 made a number of recommendations for future research that included, for example, development of models that can accommodate measurement error, missing data and multiple surrogate endpoints and/or multiple clinical outcomes. Methods for evaluating multiple surrogate endpoints were proposed by Xu and Zeger 8 for time‐to‐event data modelled jointly with multiple biomarkers measured longitudinally but were mainly limited to individual‐level data. Other examples of validating multiple surrogate endpoints include the plasma HIV‐1 RNA and CD4^+^ lymphocytes as predictors of progression to AIDS in HIV‐positive patients 9, 10 and relapse rate and number of active lesions in the brain as predictors of disability progression in relapsing remitting multiple sclerosis (RRMS) 11, which used methods that did not allow for inclusion of the measurement error for the surrogate endpoint.

Bivariate meta‐analytical methods can be used to predict the treatment effect on the target outcome from the effect on surrogate endpoint (while taking into account the uncertainty around the treatment effect on surrogate endpoint ignoring of which can impact on predictions 12) as well as to combine evidence on treatment effect on both outcomes by ‘borrowing of strength’ across outcomes when evaluating new health technologies 13, 14. Extending such methods to multivariate models can be used to evaluate multiple surrogate endpoints as joint mediators of clinical benefit with a potential advantage of increasing the precision of predictions. Multivariate meta‐analysis models require within‐study correlations between multiple effect estimates which are usually not available but need to be accounted for 13, 15. If individual patient data (IPD) are not available, but results of the Prentice's criteria for surrogacy are, then we propose to use those criteria to obtain the within‐study correlations.

Models considered in this paper build on a multivariate meta‐analysis model of mixed outcomes developed by Bujkiewicz *et al*. 16, where the between‐study covariance is parameterised in a formulation of a product of univariate normal distributions. In this model, an assumption of conditional independence between outcomes was used to simplify the model by putting a structure on the between‐study covariance matrix. Building on this model, we extend it to two alternative models. In the first case, the assumption is relaxed to allow for full unstructured covariance to be modelled in a product normal formulation, where multiple surrogate endpoints and the final outcome are correlated and hence one surrogate endpoint can also act as a surrogate to the other. In the second case, we propose an alternative parameterisation to the one by Bujkiewicz *et al*. 16, which also assumes conditional independence between some of the outcomes (by putting a structure on the between‐study covariance) but is more suitable for defining criteria for surrogacy. In both models, the product normal parameterisation offers the possibility of describing the criteria for surrogacy in a greater detail compared with providing a between‐study correlation only, which would be the case when modelling the covariance structure directly. The modelling techniques are investigated using example in RRMS, where the disability worsening is the final outcome, while relapse rate and number of active MRI lesions have been considered potentially good surrogates to the disability progression 11, 17.

In the remainder of this paper, the two models (with unstructured and structured covariance matrix) are introduced in Section 2, where in addition to this a model for obtaining the within‐study correlation from published Prentice's criteria is described. In Section 2.7, the use of software is briefly reported, which is then followed by the application of the methods to data specific to the example in RRMS which is presented together with the results in Section 3. Simulation study investigating the robustness of the methods to the normality assumption as well as the performance of the methods is described in Section 4. Extensions of the two approaches to the multivariate case are developed in Section 5. The paper concludes with a discussion of methods in Section 6.

## 2 Trivariate random effects meta‐analysis with application to surrogate endpoints

Suppose in each study *i*, we have three estimates of treatment effect observed on each of the three outcomes *Y*_1*i*_, *Y*_2*i*_ and *Y*_3*i*_, where *Y*_3_ is the treatment effect estimate for the final clinical outcome, while *Y*_1_ and *Y*_2_ are intermediate surrogate endpoints. Assuming the treatment effect estimates for the three outcomes in each study have a trivariate normal sampling distribution, the model can be written as

(1) $$\left(\begin{array}{c} Y_{1i}\\ Y_{2i}\\ Y_{3i} \end{array}\right)∼N\left(\left(\begin{array}{c} \mu_{1i}\\ \mu_{2i}\\ \mu_{3i} \end{array}\right),\Sigma_{i}\right),\;\Sigma_{i}=\left(\begin{array}{ccc} \sigma_{1i}^{2} & \sigma_{1i}\sigma_{2i}\rho_{\mathrm{wi}}^{12} & \sigma_{1i}\sigma_{3i}\rho_{\mathrm{wi}}^{13}\\ \sigma_{2i}\sigma_{1i}\rho_{\mathrm{wi}}^{12} & \sigma_{2i}^{2} & \sigma_{2i}\sigma_{3i}\rho_{\mathrm{wi}}^{23}\\ \sigma_{3i}\sigma_{1i}\rho_{\mathrm{wi}}^{13} & \sigma_{3i}\sigma_{2i}\rho_{\mathrm{wi}}^{23} & \sigma_{3i}^{2} \end{array}\right)$$

(2) $$\left(\begin{array}{c} \mu_{1i}\\ \mu_{2i}\\ \mu_{3i} \end{array}\right)∼N\left(\left(\begin{array}{c} \beta_{1}\\ \beta_{2}\\ \beta_{3} \end{array}\right),T\right),\;T=\left(\begin{array}{ccc} \tau_{1}^{2} & \tau_{1}\tau_{2}\rho_{b}^{12} & \tau_{1}\tau_{3}\rho_{b}^{13}\\ \tau_{2}\tau_{1}\rho_{b}^{12} & \tau_{2}^{2} & \tau_{2}\tau_{3}\rho_{b}^{23}\\ \tau_{3}\tau_{1}\rho_{b}^{13} & \tau_{3}\tau_{2}\rho_{b}^{23} & \tau_{3}^{2} \end{array}\right).$$

In the aforementioned model, *Y*_1*i*_, *Y*_2*i*_ and *Y*_3*i*_ are assumed to be estimates of the correlated true treatment effects *μ*_1*i*_, *μ*_2*i*_ and *μ*_3*i*_ with corresponding within‐study covariance matrices *Σ*_*i*_ of the estimates (comprising of the within‐study correlations $\rho_{\mathrm{wi}}^{\mathrm{jk}}$ between the estimates *Y*_*ji*_ and *Y*_*ki*_ and within‐study standard deviations *σ*_*ji*_ corresponding to each estimate *Y*_*j*_, for each outcome *j* = 1,2,3 and study *i*). These true study‐level effects are assumed to follow a trivariate normal distribution with means (*β*_1_,*β*_2_,*β*_3_) and covariance **T** (comprising of the between‐study correlations $\rho_{b}^{\mathrm{jk}}$ between the true treatment effects *μ*_*j*_ and *μ*_*k*_ and between‐study standard deviations *τ*_*j*_ corresponding to each true effect *μ*_*ji*_, *j* = 1,2,3 in each study *i*). In this hierarchical framework, Equations (1) and (2) describe the within‐study and the between‐study models, respectively.

In the remainder of this Section, the procedure for the use of the aforementioned model for the purpose of the validation of the surrogate endpoints is described in Section 2.1. The estimation of the variances for missing outcomes (omitted for the purpose of the validation) and the within‐study correlations (which are not reported for any of the studies but can be obtained from reported individual‐level surrogacy criteria) is described in Section 2.2. This is followed by setting out scenarios for modelling surrogate endpoints, by the use of an unstructured or structured between‐study covariance matrix, in Section 2.3 followed by the details of the modelling in the product normal formulation with the unstructured covariance matrix *T* in Section 2.4 and structured covariance in Section 2.5. The section is concluded by defining surrogacy criteria for the models in Section 2.6 and remark on the software use in Section 2.7.

### 2.1 Application of multivariate meta‐analysis to evaluating surrogate endpoints

#### 2.1.1 Using multivariate meta‐analysis to make predictions from surrogate outcomes

Traditionally, multivariate meta‐analysis is used to estimate pooled effects for multiple outcomes from multiple studies by taking into account the correlation between the outcomes. Studies included in a multivariate meta‐analysis may all report all of the outcomes, or alternatively, it is permitted that some of the studies report only a subset of outcomes under a missing at random assumption. In the latter case, the unreported outcomes can be predicted for each of the studies by taking into account the correlation between the outcomes. In a Bayesian framework, this can be achieved by coding the unreported outcomes as missing, which are then predicted by the Markov chain Monte Carlo (MCMC) simulation of the model (1)–(2) 16. This framework can be adopted to validating surrogate endpoints (at a study level).

#### 2.1.2 Using cross‐validation to examine the accuracy of predictions from multivariate meta‐analysis

The study‐level validation is conducted in the form of cross‐validation similar to the ‘leave‐one‐out’ approach as for example in Daniels and Hughes 2, except instead of taking one study out, only the estimate for the final outcome in one study is taken out. For all of the studies in the data set, one study at a time, the following procedure is conducted. For a given study, the estimate of the treatment effect on the final outcome is omitted (and coded as missing as if this particular study was a new study with the final outcome yet unknown). Then the multivariate meta‐analytic framework (1)–(2) is applied to the data including the study with the estimates of the effects on candidate surrogate endpoints known but on the final outcome missing. This missing effect is then predicted by the MCMC simulation from observed effect(s) on surrogate endpoint(s) by taking into account the data on all outcomes from the remaining studies and the relationship between the effects on all outcomes defined by the multivariate meta‐analytic model. The observed estimate is then compared with the predicted value by checking whether the value of the observed estimate falls within the predicted interval. To see if this framework will give reliable predictions for the omitted final outcome in one study, this procedure is repeated for each study *i*( i=1,…,I), giving *I* comparisons of how the predictions from the surrogate endpoints using the multivariate modelling approach perform. Ideally, we want to compare the performance of the model in predicting the true effect *μ*_3*n*_ in a new study *n*. But we do not know what the true effect is, so instead, we predict ${Ŷ}_{3n}$ and then compare the estimate *Y*_3*n*_ with the predicted interval with the variance $\sigma_{3n}^{2}+\mathrm{var}({\hat{\mu }}_{3n}|Y_{1n},Y_{2n},\sigma_{1n},\sigma_{2n},Y_{1(-n)},Y_{2(-n)},Y_{3(-n)})$, where *Y*_1(2,3)(−*n*)_ denote the data from the remaining studies without the validation study *n*.

Post the validation process, when endpoints are established as surrogate endpoints to the final outcome, this multivariate meta‐analytic framework can be used to predict treatment effect on the final outcome from those on the surrogate endpoints in a new study, for example, for the purpose of early decision making process, in particular when the treatment effect(s) on surrogate endpoint(s) is(are) measured early compared with the final outcome.

### 2.2 Obtaining within‐study variances and covariances

The within‐study variability in each study *i* is represented by the within‐study covariance matrices *Σ*_*i*_ in model (1) which are assumed to be known. In practice, only the variances can be obtained from the reported data by calculating standard errors squared, while the within‐study correlations between the treatment effect estimates are usually not reported. Also the within‐study variance of the treatment effect for the final outcome in the study in which this effect is omitted in the cross‐validation (Section 2.1) will be unknown at the validation stage. In this case, the variance will be coded as missing and a distribution has to be placed over a missing node (required if using WinBUGS for MCMC simulation). Uniform prior distribution was placed on the missing variance, $\sigma_{1,(2,3)}^{2}∼\mathrm{unif}(0.001,1000)$, which was recommended as a non‐informative prior distribution for variances by Lambert *et al* 18 (who also list other suitable prior distributions).

#### 2.2.1 Within‐study covariances

Calculation of the within‐study covariances between estimates *Y*_*ji*_ and *Y*_*ki*_, *Σ*_*i*_[*j*,*k*], for each pair of outcomes *j*,*k* in each study *i* requires knowledge of the estimate of the within‐study correlations $\rho_{\mathrm{wi}}^{\mathrm{jk}}$. These correlations between estimates of the treatment effects can be obtained by bootstrapping 2 (or double bootstrapping for correlation with uncertainty 16) of the IPD from all of the studies or a subset of studies in the meta‐analysis. Here, we consider an alternative approach where IPD is not available for any of the studies, but surrogacy on individual level has been investigated and reported by the use of Prentice's criteria.

To obtain the within‐study correlations $\rho_{\mathrm{wi}}^{\mathrm{jk}}$ between each pair of the treatment effect estimates *Y*_1*i*_, *Y*_2*i*_ and *Y*_3*i*_, calculated as a difference between two measurements in the experimental and control arms, we adopt an approach similar to the one developed by Wei and Higgins 19 who used a bivariate delta method to express the within‐study covariance between treatment effects, such as, for example, log odds ratios, in terms of the covariances between outcomes, such as the probabilities (or risks) and using a correlation between the outcomes from the literature. Here, this approach is adopted in a simpler form by representing the within‐study covariance between treatment effects in terms of the variances and correlations between the effects on specific treatment arms. In the case considered here, this is sufficient (and does not require the use of the bivariate delta method) as the correlations between effects on arm level can be obtained from Prentice's criteria of association of outcomes modelled on the absolute normal scale. However, to estimate the variances of effects in each arm, sufficient data need to be available, such as two by two table (or log odds of event with corresponding standard error) for Binomial outcomes or event rates and number of individuals (or log rates with corresponding standard errors) for Poisson outcomes.

The within‐study covariances between treatment effects on each pair of normally distributed outcomes *j*,*k* (*j* ≠ *k*;*j*,*k* = 1,2,3) can be expressed in terms of the covariances between arm‐specific effects:

(3) $$\begin{array}{cc} \Sigma_{i}[j,k]=\Sigma_{i}[k,j] & =\mathrm{Cov}\;\left(Y_{\mathrm{ji}},Y_{\mathrm{ki}}\right)\\ =\mathrm{Cov}\;\left(X_{\mathrm{eji}}-X_{\mathrm{cji}},X_{\mathrm{eki}}-X_{\mathrm{cki}}\right)\\ =\mathrm{Cov}\;\left(X_{\mathrm{eji}},X_{\mathrm{eki}}\right)-\mathrm{Cov}\left(X_{\mathrm{eji}},X_{\mathrm{cki}}\right)-\mathrm{Cov}\left(X_{\mathrm{cji}},X_{\mathrm{eki}}\right)+\mathrm{Cov}\left(X_{\mathrm{cji}},X_{\mathrm{cki}}\right)\\ =\mathrm{Cov}\;\left(X_{\mathrm{eji}},X_{\mathrm{eki}}\right)+\mathrm{Cov}\left(X_{\mathrm{cji}},X_{\mathrm{cki}}\right) \end{array}$$

where *Y*_*ji*_ (*Y*_*ki*_) is the treatment difference on outcome *j* (*k*) (such as a mean difference or log odds ratio), *X*_*aji*_ (*X*_*aki*_) are the absolute treatment effects on outcome *j* (*k*) (such as mean or log odds) in arm *a* (where e and c stand for an experimental and control arm, respectively) in study *i* and

(4) $$\mathrm{Cov}(X_{\mathrm{aji}},X_{\mathrm{aki}})=\sqrt{\mathrm{Var}(X_{\mathrm{aji}})\mathrm{Var}(X_{\mathrm{aki}})}\rho_{\mathrm{jki}}^{*}$$

where $\rho_{\mathrm{jki}}^{*}$ is the correlation between the absolute effects measured on outcomes *j* and *k* in each study *i*, which can be obtained from reported surrogacy criteria as discussed in the succeeding section.

#### 2.2.2 Within‐study correlations

Taking into account the above derivation of the within‐study covariance, the within‐study correlation can be obtain from

(5) $$\rho_{\mathrm{wi}}^{\mathrm{jk}}=\frac{\Sigma_{i}[j,k]}{\sqrt{\Sigma_{i}[j,j]\Sigma_{i}[k,k]}}=\frac{\left(\sqrt{\mathrm{Var}(X_{\mathrm{eji}})\mathrm{Var}(X_{\mathrm{eki}})}+\sqrt{\mathrm{Var}(X_{\mathrm{cji}})\mathrm{Var}(X_{\mathrm{cki}})}\right)\rho_{\mathrm{jki}}^{*}}{\sqrt{\sigma_{\mathrm{ji}}^{2}\sigma_{\mathrm{ki}}^{2}}},$$

where, as noted in the previous section, it is assumed that the variances of *X*_*aj*(*k*)*i*_ can be obtained from the data (for example from two by two tables for binary outcomes). Therefore, only the correlations $\rho_{\mathrm{jki}}^{*}$ between the effects on arm level need to be estimated in order to calculate the within‐study correlations, $\rho_{\mathrm{wi}}^{\mathrm{jk}}$, between treatment effects on each pair of outcomes *j* and *k*. Consider that *X*_*ji*_ is an average measurement on surrogate endpoint *S*_*i*_, that is, *X*_*ji*_=*E*(*S*_*i*_) and *X*_*ki*_ is an average measurement on final endpoint *F*_*i*_, that is, *X*_*ki*_=*E*(*F*_*i*_), and $\rho_{\mathrm{jki}}^{*}$ is the correlation between those average effects. Noting that for normally distributed outcomes, the correlation between the normally distributed individual observations equals the correlation between the means ensures that $\rho_{\mathrm{jki}}^{*}$ should equal the correlation between the individual‐level responses for S and F that can be obtained by modelling individual patient data.

When investigating the association on individual level between surrogate endpoint *S*_*im*_ and final outcome *F*_*im*_ for patient *m* under treatment *Z*_*im*_ in a study *i*, Prentice's criteria can be used to evaluate surrogacy. The four Prentice's criteria require that (i) there is significant treatment effect on the surrogate endpoint, (ii) there is significant treatment effect on the final outcome, (iii) surrogate endpoint has a significant impact on the final outcome and (iv) the effect of treatment on the final outcome is fully mediated by the surrogate endpoint 1, 4. Similarly as by Burzykowski, Molenberghs and Buyse 1, the first two criteria for the normally distributed outcomes can be written for each study *i* in the following model:

(6) $$\begin{array}{ccc} S_{\mathrm{im}} & = & \mu_{\mathrm{Si}}+\alpha_{i}Z_{\mathrm{im}}+\varepsilon_{\mathrm{Sim}},\\ F_{\mathrm{im}} & = & \mu_{\mathrm{Fi}}+\beta_{i}Z_{\mathrm{im}}+\varepsilon_{\mathrm{Fim}} \end{array}$$

with correlated error structure

$$\Omega =\left(\begin{array}{cc} \omega_{\mathrm{iSS}} & \omega_{\mathrm{iSF}}\\ \omega_{\mathrm{iFF}} \end{array}\right),$$

and hence, the correlation between *S*_*i*_ and *F*_*i*_, referred to by Buyse and Mollenberghs 20 as adjusted association (between *S*_*i*_ and *F*_*i*_ after adjustment for the treatment *Z*_*i*_), equals $\rho_{\mathrm{ZiSF}}=\frac{\omega_{\mathrm{iSF}}}{\sqrt{\omega_{\mathrm{iSS}}\omega_{\mathrm{iFF}}}}$. This is the correlation in the individual‐level response for S and F which as noted above equals the correlation between the means, in this case between *E*(*S*_*im*_) and *E*(*F*_*im*_) and hence between *X*_*ji*_ and *X*_*ki*_. Thus the correlation *ρ*_*ZiSF*_ equals the correlation $\rho_{\mathrm{jki}}^{*}$ between the absolute effects in (4) if, for example, *j* = *S* and *k* = *F*. Also the average treatment effects *α*_*i*_ and *β*_*i*_ on the two outcomes are the treatment effect estimates *Y*_*ji*_ and *Y*_*ki*_ in study *i*. The correlation *ρ*_*ZiSF*_ is required to estimate the within‐study correlation $\rho_{\mathrm{wi}}^{\mathrm{jk}}$, which can be obtained from the fourth Prentice's criteria 4 which Burzykowski, Molenberghs and Buyse 1 propose to verify through the conditional distribution of the final outcome conditional on the treatment and the surrogate endpoint,

(7) $$F_{\mathrm{im}}={\tilde{\mu }}_{\mathrm{iF}}+\beta_{\mathrm{iS}}Z_{\mathrm{im}}+\gamma_{\mathrm{iZ}}S_{\mathrm{im}}+{\tilde{\varepsilon }}_{\mathrm{Fim}},$$

where *β*_*iS*_=*β*_*i*_−*α*_*i*_ *ω*_*iFS*_/*ω*_*iSS*_ and *γ*_*iZ*_=*ω*_*iFS*_/*ω*_*iSS*_. This criterion requires that if all treatment effect is mediated by the surrogate endpoint *S* then *β*_*iS*_ should be zero. Rearranging the terms for *β*_*iS*_ gives *ω*_*iFS*_=*ω*_*iSS*_(*β*_*i*_−*β*_*iS*_)/*α*_*i*_, which substituted into the formula for the adjusted association *ρ*_*ZiSF*_ gives

(8) $$\rho_{\mathrm{ZiSF}}=\frac{\beta_{i}-\beta_{\mathrm{iS}}}{\alpha_{i}}\sqrt{\omega_{\mathrm{iSS}}/\omega_{\mathrm{iFF}}}.$$

The parameters *α*_*i*_, *β*_*i*_, *ω*_*iSS*_ and *ω*_*iFF*_ can be obtained by fitting model (6) to the patient data on responses *S* and *F*.

To complete the individual‐level validation, the third Prentice's criteria can be verified by

(9) $$F_{\mathrm{im}}=\mu_{i}+\gamma_{i}S_{\mathrm{im}}+\varepsilon_{\mathrm{im}},$$

with further details discussed by Burzykowski, Molenberghs and Buyse 1.

In the trivariate meta‐analysis, the within‐study correlations between treatment effects need to be estimated for each pair of outcomes. To do this using the above criteria, one of the outcomes in each pair is considered a surrogate endpoint to other outcome. Ideally, the within‐study correlations (or the Prentice's criteria from which the correlations derive) should be obtained from the IPD from each study in the meta‐analysis. When IPD is available from each of the studies, the within‐study correlation can be calculated directly. However, in the absence of the data, it may be possible to obtain the Prentice's criteria corresponding to the data from each (or some) of the studies (or some of the studies in the meta‐analysis). The Prentice's criteria provide both the assessment of surrogacy on the individual level as well as sufficient information to obtain the within‐study correlations. This approach is illustrated in Section 3, where the Prentice's criteria published in literature for one of the studies included in the meta‐analysis for the motivating example in RRMS are used to estimate the within‐study correlation. Note that establishing the within‐study correlation does not guarantee causal relationship between the treatment effects on surrogate and final outcomes. Research developments on causality of surrogate endpoints are highlighted in the Discussion section.

### 2.3 Scenarios for modelling of surrogate endpoints

**Figure 1:**
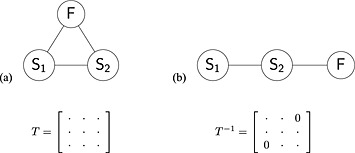
Scenarios for modelling surrogates endpoints: (a) all outcomes correlated giving unstructured covariance matrix *T*, (b) final outcome conditionally independent from the first surrogate endpoint conditional on the second giving structured covariance matrix equivalent to the precision matrix *T* ^−1^ with element [1,3] equal to zero.

Two scenarios for modelling the surrogate endpoints are considered here. In the first scenario, true treatment effects, *μ*_*ji*_, on all the outcomes are assumed correlated as shown in Figure 1a. In this case, the between‐study covariance matrix *T* in the model (2) is unstructured and can be modelled directly, by either placing inverse Wishart prior distribution on *T*, by Cholesky or spherical decomposition 21 (and placing separate prior distributions on the between‐study correlations and standard deviations) or by re‐parameterising the between‐study model in the product normal formulation of the series of univariate conditional distributions. The latter approach has a number of advantages. In contrast to modelling it directly by the use of Wishart prior distribution, the product normal formulation allows direct control over the prior distributions on all elements of the between‐study covariance matrix (between‐study standard deviations and correlations). It also allows to describe some association criteria which helps to assess the surrogacy in more detail compared with obtaining only between‐study correlation.

In the second scenario, shown in Figure 1b, in which the treatment effects on multiple outcomes may (but do not have to) be assumed to be measured sequentially in time, assumption is made that the treatment effect on the final outcome is conditionally independent from the effect on the first surrogate endpoint conditional on the effect on the second surrogate. This assumption puts a structure on the between‐study covariance matrix *T* resulting in element [1,3] of the precision matrix *T* ^−1^ being equal to zero. This approach leads to a reduced number of parameters to estimate and is easier to implement, in particular when dealing with multiple outcomes beyond the trivariate case (for details see Sections 5.1 and 5.2).

### 2.4 Product normal formulation with unstructured covariance matrix

For the first scenario of the true treatment effects *μ*_*ji*_ on all outcomes being correlated, represented graphically in Figure 1a, the between‐study covariance has an unstructured form. The between study model (2) for this scenario is re‐parameterised in the product normal formulation of the series of univariate conditional distributions:

(10) $$\left\{\begin{array}{c} \mu_{1i}∼N\left(\eta_{1},\psi_{1}^{2}\right)\\ \mu_{2i}|\mu_{1i}∼N\left(\eta_{2i},\psi_{2}^{2}\right)\\ \eta_{2i}=\lambda_{20}+\lambda_{21}\mu_{1i}\\ \mu_{3i}|\mu_{1i},\mu_{2i}∼N\left(\eta_{3i},\psi_{3}^{2}\right)\\ \eta_{3i}=\lambda_{30}+\lambda_{31}\mu_{1i}+\lambda_{32}\mu_{2i}. \end{array}\right.$$

Instead of placing independent non‐informative prior distributions on all the parameters and hyperparameters of the model, relationships between these parameters and the elements of the between‐study covariance matrix are derived to allow to take into account the inter‐relationship between the parameters. These relationships have the following forms:

(11) $$\begin{array}{cc} \psi_{1}^{2} & =\tau_{1}^{2},\;\;\psi_{2}^{2}=\tau_{2}^{2}-\lambda_{21}^{2}\tau_{1}^{2},\;\;\psi_{3}^{2}=\tau_{3}^{2}-\lambda_{31}^{2}\tau_{1}^{2}-\lambda_{32}^{2}\tau_{2}^{2},\\ \lambda_{21} & =\frac{\tau_{2}}{\tau_{1}}\rho_{b}^{12},\;\;\lambda_{31}=\rho_{b}^{13}\frac{\tau_{3}}{\tau_{1}}-\lambda_{32}\lambda_{21},\;\;\lambda_{32}=\left(\rho_{b}^{23}\tau_{2}\tau_{3}-\rho_{b}^{31}\tau_{3}\tau_{1}\lambda_{21}\right)/\left(\tau_{2}^{2}-\lambda_{21}^{2}\tau_{1}^{2}\right) \end{array}$$

which are obtained by following the procedure described in detail in Section 5.1 for *N*‐dimensional case. Having established them allows to place prior distributions on the between‐study standard deviations and correlations, which is an easier task as the plausible range of values for these parameters are known or can be obtained from external sources of information (see, for example, Higgins and Whitehead 22 or Bujkiewicz *et al*. 16). By placing prior distributions on these parameters, for example, $\rho_{b}^{12(13,23)}∼\mathrm{dunif}(-1,1)$, *τ*_1(2,3)_∼*N*(0,10)*I*(0,) (normal distribution truncated at value zero), the above derived relationships give the implied prior distribution on the parameters *λ*_21_, *λ*_31_ and *λ*_32_ and hyper‐parameters *ψ*_1_, *ψ*_2_ and *ψ*_3_. The remaining parameters are given ‘vague’ prior distributions, *η*_1_∼*N*(0,1000), *λ*_20(30)_∼*N*(0,1000).Note that the pooled effects *β*_1(2,3)_ on the three outcomes in model (2) are also directly linked to the model (10); *β*_1_=*η*_1_, *β*_2_=*λ*_20_+*λ*_21_ *β*_1_ and *β*_3_=*λ*_30_+*λ*_31_ *β*_1_+*λ*_32_ *β*_2_. It is possible to center the true effects on surrogate endpoints (by replacing *μ*_*ji*_ with $\left(\mu_{\mathrm{ji}}-\bar{\mu_{\mathrm{ji}}}\right)$ in the third and fifth line of formula (10), which can be useful if there are problems with autocorrelation) in which case the intercepts would equal the pooled effects; *β*_2_=*λ*_20_ and *β*_3_=*λ*_30_.

### 2.5 Product normal formulation with structured covariance matrix

A simplified model can be used by assuming conditional independence between treatment effects on some of the outcomes. For example, we can consider a situation where treatment effect is measured on different outcomes sequentially in time (or on the same outcome repeatedly in time). For example, if treatment effect on first outcome is measured at 12 months, second at 24 months and third at 36 months, then it may be conceivable to assume that the effect on the final outcome may be conditionally independent from the effect on the first outcome conditional on the second. This scenario, described in Figure 1b, leads to a simplified between‐study model with the true effect on the final outcome now conditional on the effect on the second surrogate endpoint only:

(12) $$\left\{\begin{array}{c} \mu_{1i}∼N\left(\eta_{1},\psi_{1}^{2}\right)\\ \mu_{2i}|\mu_{1i}∼N\left(\eta_{2i},\psi_{2}^{2}\right)\\ \eta_{2i}=\lambda_{20}+\lambda_{21}\;\mu_{1i}\\ \mu_{3i}|\mu_{2i}∼N\left(\eta_{3i},\psi_{3}^{2}\right)\\ \eta_{3i}=\lambda_{30}+\lambda_{32}\;\mu_{2i}, \end{array}\right.$$

The assumption of conditional independence of the true treatment effects on outcomes three and one, *μ*_3_ and *μ*_1_, conditional on the effect on outcome two, *μ*_2_ puts a structure on the covariance matrix giving the corresponding element ({1,3}) of the inverse covariance (precision) matrix equal to zero. This means that partial correlation $\rho_{b}^{13|2}=0$. Because the partial correlation between *μ*_1_ and *μ*_3_ (adjusted for *μ*_2_) equals $\rho_{b}^{13|2}=\left(\rho_{b}^{13}-\rho_{b}^{12}*\rho_{b}^{23}\right)/\sqrt{1-{\left(\rho_{b}^{12}\right)}^{2}}\sqrt{1-{\left(\rho_{b}^{23}\right)}^{2}}=0$, it implies that $\rho_{b}^{13}=\rho_{b}^{12}*\rho_{b}^{23}$. This reduces the number of parameters in the model that need to be estimated and also simplifies the relationships between the parameters and hyperparameters of the model with the elements of the between‐study covariance matrix:

(13) $$\begin{array}{cc} \psi_{1}^{2} & =\tau_{1}^{2},\;\;\psi_{2}^{2}=\tau_{2}^{2}-\lambda_{21}^{2}\tau_{1}^{2},\;\;\psi_{3}^{2}=\tau_{3}^{2}-\lambda_{32}^{2}\tau_{2}^{2},\\ \lambda_{21} & =\rho_{b}^{12}\frac{\tau_{2}}{\tau_{1}},\;\;\lambda_{32}=\rho_{b}^{23}\frac{\tau_{3}}{\tau_{2}}, \end{array}$$

which both can have an advantage in scenarios with multiple outcomes beyond trivariate case as discussed in Section 5. Similarly as in the case of the full unstructured covariance matrix, placing prior distributions directly on the between‐study standard deviations *τ*_1(2,3)_∼*N*(0,10)*I*(0,) and correlations $\rho_{b}^{12(23)}∼\mathrm{dunif}(-1,1)$ gives implied prior distributions placed on the parameters of the model (12), *ψ*_1(2,3)_ and *λ*_21(32)_ obtained from the the derived relationship between the two sets of parameters. The remaining parameters are given non‐informative prior distributions *η*_1_∼*N*(0,1000), *λ*_20(30)_∼*N*(0,1000). The pooled effects *β*_1(2,3)_ on the three outcomes in model (2) are also directly linked to the model (12); *β*_1_=*η*_1_, *β*_2_=*λ*_20_+*λ*_21_ *β*_1_ and *β*_3_=*λ*_30_+*λ*_32_ *β*_2_. As in the model in Section 2.4, centering of the true effects on surrogate endpoints can be applied to this models in which case the intercepts would equal the pooled effects.

Assuming such sequential structure of the treatment effects, that could be measured on the same outcome at multiple time points, the model can lead to removing some of the measurement error, for example at time two using time one. We cannot measure the true effect *μ*_2*i*_ at time two (only have an estimate *Y*_2*i*_), so having measurement at time one can improve estimate of measurement at time two. This can be useful in particular when the treatment effect at time one is measured precisely and at time two inaccurately, then the prediction at time two may be more precise (due to accounting for the correlation with outcome one) leading to better prediction at time three.

### 2.6 Criteria for surrogate markers

Consider first a bivariate case (with one surrogate endpoint, where the first endpoint is surrogate to the second and the third outcome is removed) as described in the first three lines of Equation (10) or (12). We can then follow the criteria set out by Daniels and Hughes 2, by which *λ*_21_ indicates the association between the treatment effect measured by the surrogate endpoint and the treatment effect measured by the second clinical outcome (final outcome in the bivariate case with a single surrogate endpoint), therefore, we require *λ*_21_≠0. For the association to be perfect, the conditional variance should be zero; $\psi_{2}^{2}=0$. Also, we would expect *λ*_20_=0 (no treatment effect on the surrogate endpoint gives no treatment effect on the target outcome), otherwise not all of the treatment effect on the target outcome is mediated by the effect on the surrogate endpoint. In the trivariate case with two surrogate markers, the effects of treatment on all biomarkers may jointly mediate the treatment effect on the final outcome. For the combined effect on the biomarkers to fully mediate the effect on the target outcome, we expect the intercept *λ*_30_=0 and the conditional variance $\psi_{3}^{2}=0$. The association between the effect on target outcome and each of the surrogate endpoints is expected not to be zero; *λ*_31,32_≠0. In the sequential scenario of conditionally independent effects (described in Section 2.5), the same criteria as in the bivariate case apply, where the effect on the first outcome is a surrogate to the effect on the second and the effect on the second outcome is a surrogate to the effect on the third (final) outcome, but with additional ‘borrowing of strength’ across outcomes by taking into account the correlation structure between all of the outcomes (i.e. only conditional independence assumed).

### 2.7 Implementation in WinBUGS and R

All models were implemented in WinBUGS 23 where the estimates were obtained using MCMC simulation using 50000 iterations (including 20000 burn‐in). Convergence was checked by visually assessing the history, chains and autocorrelation using graphical tools in WinBUGS. All posterior estimates are presented as means with the 95% credible intervals (CrI). R was used for data manipulation and to execute WinBUGS code multiple times (for validation of surrogates for each study) using the R2WinBUGS package 24. OpenBUGS and R2OpenBUGS version of the software was used for the simulation study which was conducted using Linux (Red Hat, Inc., Raleigh, North Carolina)‐based high performance computer.

WinBUGS programs corresponding to the two TRMA models (applied to data in Tables 1 and 2) are included in Web Supplements A1 and A2.

**Table 1:** Data on disability progression, relapse rate and number of active MRI lesions included in the meta‐analysis.

		Disability progression					Relapse rate					MRI			
**Study**	Follow‐up (months)	*N* *d*_*E*_	*R* *d*_*E*_	*N* *d*_*C*_	*R* *d*_*C*_		*N* *r*_*E*_	*A* *R* *r*_*E*_	*N* *r*_*C*_	*A* *R* *r*_*C*_		*N* *m*_*E*_	*R* *m*_*E*_ (SE)	*N* *m*_*C*_	*R* *m*_*C*_ (SE)
Paty (A)	24	124	35	124	35		124	145	124	157		124	1.80 (0.40)	124	4.9 (1.30)
Paty (B)	24	124	25	124	35		124	104	124	157		124	2.00 (0.70)	124	4.9 (1.30)
Jacobs	24	85	18	87	29		85	52	87	78		85	3.20 (0.41)	87	4.8 (0.49)
Millefiorini	24	27	2	24	9		27	12	24	31		23	3.50 (0.71)	19	7.3 (1.84)
Li (C)	24	189	57	187	69		189	172	187	240		189	9.00 (4.00)	187	15.5 (2.90)
Li (D)	24	184	50	187	69		184	159	187	240		184	5.50 (0.50)	187	15.5 (2.90)
Polman	24	627	107	315	91		627	144	315	230		627	1.90 (0.37)	315	11.0 (0.88)
Comi (E)	24	433	62	437	90		433	61	437	144		433	0.38 (0.07)	437	1.43 (0.06)
Comi (F)	24	456	69	437	90		456	68	437	144		456	0.33 (0.06)	437	1.43 (0.06)
Rudick	24	589	135	582	169		589	200	582	437		589	0.90 (0.09)	582	5.4 (0.36)
Sorensen	24	66	11	64	16		66	15	64	38		66	2.70 (0.46)	64	3.5 (0.51)
Clanet	36	400	148	402	149		400	324	402	310		400	8.00 (0.88)	402	9.0 (0.74)
Mikol	24	386	45	378	33		386	116	378	110		172	0.58 (0.11)	178	0.77 (0.18)

*N* *d*_*E*_ (*N* *d*_*C*_), total number of patients in experimental (control) arm with disability status recorded.

*R* *d*_*E*_ (*R* *d*_*C*_), number of patients in experimental (control) arm who progressed.

*N* *r*_*E*_ (*N* *r*_*C*_), total number of patients in experimental (control) arm with number of relapses recorded.

*A* *R* *r*_*E*_ (*A* *R* *r*_*C*_), number of relapses per year in experimental (control) arm.

*N* *m*_*E*_ (*N* *m*_*C*_), total number of patients in experimental (control) arm with number of active MRI lesions in experimental (control) arm.

*R* *m*_*E*_ (*R* *m*_*C*_), mean number (standard error) of active MRI lesions in experimental (control) arm.

**Table 2:** Indicators of individual‐level surrogacy for number of active MRI lesions and relapse rate as surrogate endpoints to disablity progression (reproduced from Sormani *et al* 11) and number of active MRI lesions as a surrogate endpoint to relapse rate (reproduced from Sormani *et al* 27).

		Prentice's criteria		
Surrogate endpoint	Final outcome	1st criterion^a^	2nd criterion^b^	4th criterion^c^
active T2 lesions	disability progression	*α*_1_=−0.93(0.12)	*β*_1_=−0.37(0.19)	*β*_*S*1_=−0.14(0.19)
relapses	disability progression	*α*_2_=−0.44(0.08)	*β*_2_=−0.37(0.19)	*β*_*S*2_=−0.15(0.20)
active T2 lesions	relapses	*α*_3_=−0.90(0.13)	*β*_3_=−0.36(0.08)	*β*_*S*3_=−0.17(0.09)

Coefficients are reported with standard errors.

a ^∗^1st Prentice's criterion, treatment is effective on surrogate endpoint.

b ^*†*^2nd Prentice's criterion, treatment is effective on final clinical outcome.

c ^*‡*^4th Prentice's criterion, treatment effect on final clinical outcome fully mediated by surrogate.

*α*_1_, treatment effect on log number of MRI lesions over 1 year; *α*_2_, treatment effect on log relapse rate

over 1 year; *α*_3_, treatment effect on log number of MRI lesions over 2 years; *β*_1_,*β*_2_, treatment effect on

log odds disability progression over 2 years; *β*_3_, treatment effect on log relapse rate over 2 years.

*β*_*S*1_ and *β*_*S*2_, treatment effect on log odds disability progression over 2 years adjusted for treatment effect on

log number of MRI lesions and log relapse rate, respectively; *β*_*S*3_, treatment effect on log relapse rate

over 2 years adjusted for treatment effect on log number of MRI lesions.

## 3 Application: relapsing remitting multiple sclerosis

To illustrate the application of the methods described in Section 2, the models developed there are applied to the motivating example in RRMS described in Section 3.1. Section 3.2 (along with the Appendix A) contains details of how data in Table 1 are used to populate the models for outcomes specific to this motivating example. Results are presented in Section 3.3.

### 3.1 Introduction to the motivating example

To illustrate the use of the modelling techniques, we applied them to an example in RRMS. Multiple sclerosis (MS) is an inflammatory disease of the brain and spinal cord. RRMS is a most common type of MS. During the course of the disease, patients experience a series of periods of exacerbations (relapses) and remission. A large proportion of patients (25%) eventually progresses to secondary progressive disease 25. The disability progression is considered the final outcome, whereas the number of active (new or enlarging) T2 lesions in the brain obtained from the magnetic resonance imaging (MRI) and the annualised relapse rate are two potential surrogate endpoints. This example is based on work by Sormani *et al*. 17 who used meta‐analytic approach to evaluate whether the effects on relapse rate and number of active MRI lesions are good predictors of the treatment effects on disability progression (one surrogate endpoint at a time). In another paper, Sormani *et al*. 26 investigated estimates of the treatment effect on number of active MRI lesions as predictors of the effects on relapse rate. Subsequently, Sormani and colleagues used IPD to investigate individual‐level association between outcomes, where they validated the number of MRI lesions as a surrogate to the number of relapses 27. In another paper, Sormani *et al*. also used IPD to validate both the number of active MRI lesions and the number of relapses as surrogate endpoints to the disability progression, as individual surrogates as well as joint mediators of the treatment effect on progression 11. In the papers mentioned previously, the meta‐analytic work on study level was conducted using weighted linear regression 17, 26, whereas the association on the individual level was conducted using Prentice's criteria 11, 27.

**Figure 2:**
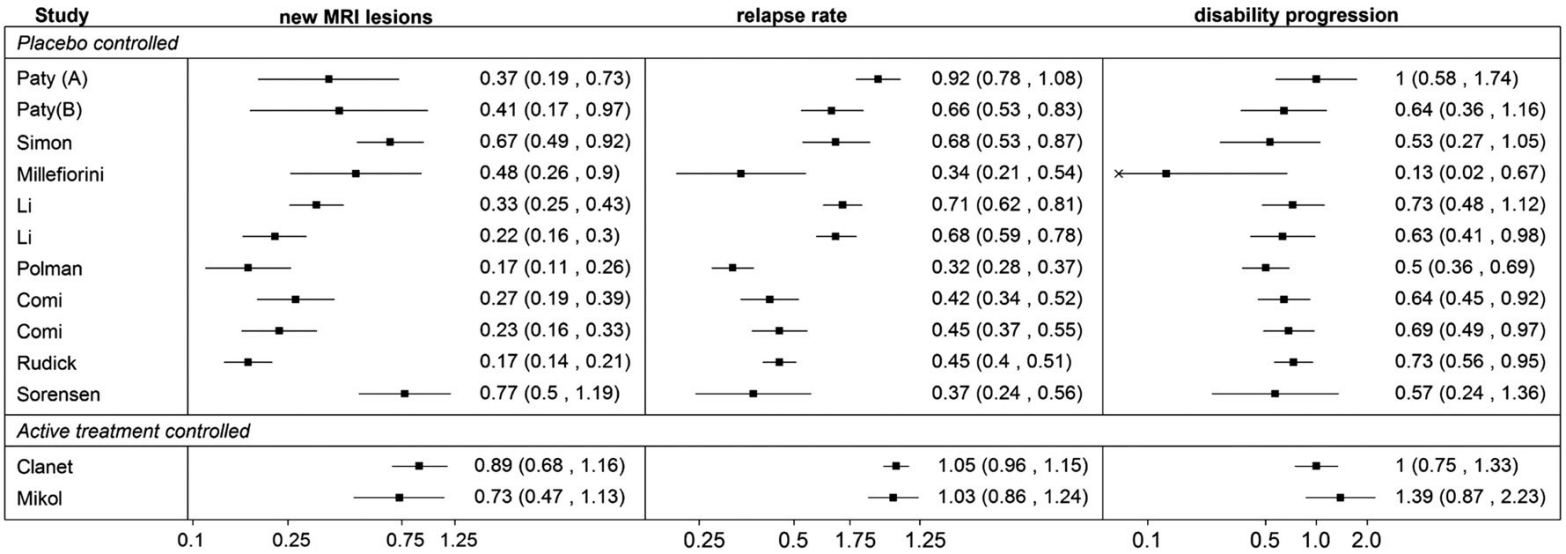
Graphical representation of data for treatment effects on MRI (surrogate endpoint 1), relapse rate (surrogate endpoint 2) and disability progression (final clinical outcome).

This example with two surrogate endpoints, where one of the candidate surrogate endpoints (to the final outcome) is also a potential surrogate endpoint to the second surrogate, serves as a desirable illustration of modelling techniques investigated in this paper. We collect data on the treatment effects on those outcomes from studies included in the analysis by Sormani *et al*. 17. To investigate the surrogate endpoints jointly, we chose to include only the studies reporting treatment effect estimates on all of the three outcomes and those that reported sufficient information to obtain uncertainty estimates around the observed treatment effect. As a result, we obtained data from 13 studies, 11 placebo‐controlled trials and two active‐controlled trials, which are listed in Table 1 and displayed graphically in Figure 2. To obtain the within‐study correlations between the treatment effect estimates for the outcomes, we use the estimates of Prentice's criteria, reported by Sormani *et al*. 27 for the association between the number of MRI lesions and the number of relapses who adopted the model (6) to those outcomes on log scale and for the association of both the number of MRI lesions and the relapses with the disability progression reported by Sormani *et al.*, 11, also on log scale. Relevant estimates are listed in Table 2. Because of limitation of the data, in this paper, the correlation is assumed constant across studies.

### 3.2 Scale of the outcomes and the within‐study model

The relative treatment effects on each outcome (MRI, relapse rate and disability progression) and the within‐study variances for the treatment effects in each study are calculated using data listed (and notation described) in Table 1.

The MRI effect is modelled on the log rate ratio (RR) scale, *Y*_1*i*_= log*M* *R* *I* *R* *R*= log(*R* *m*_*E*_/*R* *m*_*C*_). The relative treatment effect on relapses is modelled by the log annualised relapse rate ratio (ARRR) scale, $Y_{2i}=\log\mathrm{ARRR}=\log\left(\frac{\mathrm{AR}R_{E}}{\mathrm{AR}R_{C}}\right)$. The relative treatment effect on disability progression is modelled on the log odds ratio (OR) scale, $Y_{3i}=\log\mathrm{OR}=\log\left(\frac{Rd_{E}(Nd_{C}-Rd_{C})}{Rd_{C}(Nd_{E}-Rd_{E})}\right)$.

The corresponding variances are obtained from the data by the use of delta method for the effects on MRI and relapses and using the standard formulae for the standard error of log*O* *R*, as shown in Appendix A.1. The within‐study correlations are obtained following methods described in Section 2.2, with details of the algebra for the data in RRMS in Appendix A.2. The correlations were obtained for one study and assumed the same in all the studies.

Note that some authors used relative risk to quantify the treatment effect on the disability progression. However, log OR scale is applied here in order to combine the summary data on effectiveness with reported Prentice's criteria, which were modelled for the progression on the log odds scale. Typically, progression is reported cross‐sectionally as a number (or proportion) of patients who progressed (i.e. whose expanded disability status scale score had increased by at least one point at the follow up). As such, the reported outcome is not suitable to model as time to event outcome on the hazard ratio scale.

### 3.3 Results

Applying the aformentioned formulae to the RRMS data in Table 1 and combining it with data of individual‐level surrogacy criteria in Table 2 gives the within‐study correlations: $\rho_{\mathrm{wi}}^{12}=0.25$, $\rho_{\mathrm{wi}}^{13}=0.09$, $\rho_{\mathrm{wi}}^{23}=0.09$. The data are applied using the above models to validate surrogate endpoints as predictors of the clinical benefit on the final outcome using a cross‐validation procedure as described in Section 2.1. First, the models (1) and (10), assuming that all true effects are correlated, is applied. Results of this application are presented in Section 3.3.1. Then the models (1) and (12), assuming the conditional independence between the effects on the final outcome and the first surrogate endpoint is applied and results presented in Section 3.3.2.

#### 3.3.1 Results from the model with unstructured between‐study covariance matrix

To quantify the surrogacy criteria overall, the model was first applied to the full data (no outcome missing for any of the studies). The results (parameters with the 95% CrIs) are shown in Table 3 (top). The coefficients *λ*_20_ and *λ*_21_ and variance $\psi_{2}^{2}$ represent the association between the treatment effect on the number of active MRI lesions and the effect on the relapse rate, whereas the coefficients *λ*_30_, *λ*_31_, *λ*_32_ and $\psi_{3}^{2}$ describe how the effects on number of MRI lesions and relapse jointly mediate the effect on the disability progression (as described in Section 2.6). The parameters on the left‐hand‐side indicate that there was a poor association between the effects on MRI and effects on relapses, as the interval of the slope contains zero and the variance $\psi_{3}^{2}$ appears greater than zero. The parameters on the right‐hand‐side suggest that the association between the effects on both surrogate endpoints (MRI and relapse rates) are not strongly associated with the effect on disability progression (slopes contain zero), however, the variance appears small suggesting that the effect on MRI and relapse rate largely mediate the effect of the treatment on disability progression. In both cases, the CrIs for the intercepts contain zero, which is encouraging in the sense that zero treatment effect on MRI would be expected to predict zero effect on relapse rate in the first case and the zero effects measured by the MRI and relapse rate would lead to predicted zero effect on the disability progression in the second case. However, in both cases the CrIs are wide, hence, the predictions may not be accurate.

**Table 3:** Surrogacy criteria obtained from the trivariate models applied to the full data (no missing outcomes).

Parameter	Mean (95% CrI)	Parameter	Mean (95% CrI)
Unstructured between‐study covariance			
*λ*_20_	−0.28 (−0.73, 0.16)	*λ*_30_	−0.09 (−0.38, 0.21)
*λ*_21_	0.26 (−0.12, 0.65)	*λ*_31_	0.00 (−0.38, 0.31)
		*λ*_32_	0.43 (−0.02, 1.06)
$\psi_{2}^{2}$	0.15 (0.05, 0.37)	$\psi_{3}^{2}$	0.02 (0.00, 0.10)
Structured between‐study covariance			
*λ*_20_	−0.24 (−0.70, 0.20)	*λ*_30_	−0.06 (−0.31, 0.19)
*λ*_21_	0.30 (−0.09, 0.70)	*λ*_32_	0.48 (0.11, 0.88)
$\psi_{2}^{2}$	0.15 (0.05, 0.36)	$\psi_{3}^{2}$	0.02 (0.00, 0.10)

After estimating the surrogacy criteria using the full data, the cross‐validation process was carried out. The results of the validation are presented in Table 4 (column four, along with the results from the bivariate model and trivariate with structured covariance matrix), which shows the values of observed effects on the final outcome in each study (column two) and the effects on the final outcome predicted by the model from the effects on the surrogates (given full data on all other studies) with the 95% CrIs. In each row, the values correspond to the validation of prediction for a study whose corresponding observed effect on the final outcome is in the second column. In the take‐one‐out approach of the cross‐validation procedure, the regression parameters (the intercepts, slopes and conditional variances, as in the between‐study model (10)) did not change substantially. The full set of those values is included in the Web Supplement B. The predicted intervals contain the observed value of the treatment effect on the final outcome for all of the studies confirming good fit of the model.

**Table 4:** Comparison of results of validation obtained from the two trivariate models and a bivariate model.

		Predicted				
Study	Observed	Bivariate	Trivariate Unstructured	% red.	Trivariate Structured	% red.
Paty (A)	1.00 (0.57, 1.74)	0.86 (0.42,1.74)	0.86 (0.43, 1.73)	0.67	0.88 (0.44, 1.73)	3.16
Paty (B)	0.64 (0.36, 1.16)	0.76 (0.37, 1.56)	0.77 (0.38, 1.54)	2.24	0.77 (0.38, 1.54)	3.29
Simon	0.53 (0.27, 1.06)	0.78 (0.36, 1.73)	0.80 (0.36, 1.77)	0.19	0.79 (0.37, 1.72)	2.10
Millefiorini	0.13 (0.02, 0.67)	0.62 (0.11, 3.46)	0.65 (0.12, 3.62)	−0.29	0.62 (0.11, 3.44)	0.32
Li (C)	0.73 (0.47, 1.12)	0.79 (0.43, 1.45)	0.79 (0.45, 1.41)	4.46	0.80 (0.45, 1.42)	5.19
Li (D)	0.63 (0.41, 0.98)	0.79 (0.43, 1.44)	0.81 (0.44, 1.50)	−1.07	0.79 (0.45, 1.39)	7.23
Polman	0.50 (0.36, 0.69)	0.59 (0.32, 1.11)	0.61 (0.35, 1.07)	9.67	0.58 (0.33, 1.03)	8.62
Comi (E)	0.64 (0.45, 0.92)	0.62 (0.35, 1.11)	0.62 (0.36, 1.08)	3.71	0.62 (0.36, 1.07)	5.13
Comi (F)	0.69 (0.49, 0.97)	0.64 (0.36, 1.13)	0.64 (0.38, 1.09)	5.77	0.63 (0.37, 1.08)	5.93
Rudick	0.73 (0.56, 0.95)	0.62 (0.37, 1.05)	0.61 (0.37, 1.01)	4.18	0.61 (0.38, 0.98)	10.01
Sorensen	0.57 (0.24, 1.36)	0.62 (0.24, 1.64)	0.64 (0.23, 1.82)	−7.52	0.63 (0.24, 1.66)	0.63
Clanet	1.00 (0.75, 1.33)	0.89 (0.49, 1.63)	0.88 (0.49, 1.58)	3.21	0.92 (0.53, 1.60)	8.30
Mikol	1.39 (0.87, 2.23)	0.82 (0.44, 1.55)	0.83 (0.45, 1.53)	2.65	0.85 (0.46, 1.57)	3.45
average % reduction in CrI				2.14%		4.87%
DIC			350.1		347.9	

The % red refers to the percentage reduction in the width of the credible interval corresponding to the prediction from the trivariate model, with the unstructured (columns 4 and 5) or structured (columns 6 and 7) between‐study covariance matrix, compared with the width of the interval corresponding to the prediction from the bivariate model (column 3).

DIC, deviance information criteria.

#### 3.3.2 Results from the model with structured between‐study covariance matrix

Models (1) and (12) with structured between‐study covariance matrix, where the effect on the disability progression (the final outcome) is conditionally independent from the effect on the MRI (the first surrogate endpoint) conditional on the effect on relapse rate (the second surrogate endpoint) is now used for validation. Similarly as in the case of the model with unstructured covariance matrix, the surrogacy criteria are estimated using the full data, which is then followed by the cross‐validation. In Table 3 (bottom) the parameters are shown together with the 95% CrIs for the model applied to the full data. The parameters *λ*_20_, *λ*_21_ and $\psi_{2}^{2}$ describe the association between the treatment effects on the MRI and the relapse rate, while *λ*_30_, *λ*_31_ and $\psi_{3}^{2}$ the association between the treatment effects on the relapse rate and the disability progression (conditional on the effect on MRI). The association between the effects on the MRI and the relapse rate is not strong which is indicated by the interval of *λ*_21_ containing zero and the variance $\psi_{2}^{2}$ significantly larger than zero. However, the association between the effect on the relapse rate, as a surrogate endpoint, with the effect on the disability progression, as the final outcome, (conditional on the effect on MRI) appears strong as indicated by the non‐zero slope *λ*_31_ and the small variance $\psi_{3}^{2}$. Also the interval of the intercept *λ*_30_ containing zero indicates that zero effect on the relapse rate is likely to imply zero effect on the disability progression. In the take‐one‐out approach of the cross‐valdation procedure, the regression parameters (the intercepts, slopes and conditional variances, as in the between‐study model (12)) did not change substantially. The full set of those values is included in the Web Supplement B. Table 4 shows the results of cross‐validation (with column six corresponding to predictions from the model with structured between‐study covariance). The results of predictions are similar to those obtained from the model with unstructured between‐study covariance matrix.

#### 3.3.3 Comparison of the results from the two models and those from a bivariate model

**Figure 3:**
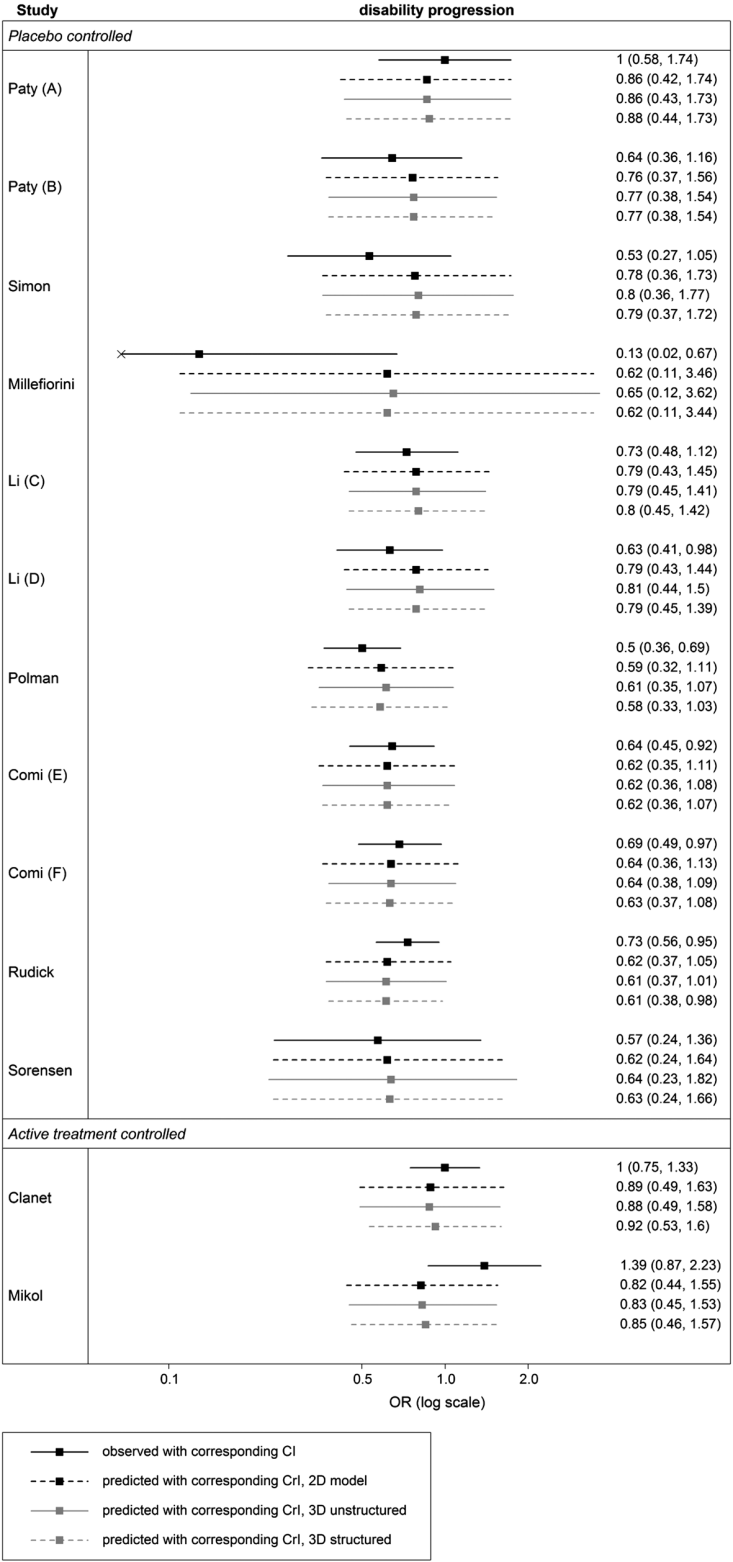
Forest plot, showing for each study the observed value of the OR of disability progression with corresponding confidence interval (CI) and the predicted values with corresponding credible intervals (CrIs) from a bivariate model, trivariate model with unstructured covariance matrix and from the trivariate model with structured covariance matrix.

When carrying out the cross‐validation process, we want to ensure that not only predicted CrIs contain the actual observed values but also that the intervals are narrow. Inclusion of multiple surrogate endpoints can potentially lead to reduced intervals and hence better predictions. To compare the aforementioned results of the validation from the two trivariate models, they are shown side by side in Table 4 as well as graphically in Figure 3 alongside those from a bivariate model with the effect on the relapse rate as a surrogate for the effect on the disability progression. In Table 4, apart from the predicted values, the percentage reduction in the width of the credible interval relative to the width of the interval obtained from the bivariate model is shown for both trivariate models. On average, the model with the unstructured between‐study covariance matrix gave intervals 2% narrower than those from the model using a single surrogate endpoint, whereas the model with the structured covariance matrix led to 5% reduction in uncertainty. Although the average gain in precision is modest, the inclusion of both surrogates does improve the predictions and this reduction in uncertainty may be sufficient to improve the decision making process based on such predictions. This may be the case in particular for larger studies, such as by Polman, Rudick and Clanet in our illustrative example, where the gain in precision was also larger, up to 10%, likely due to the treatment effect on the surrogate endpoints being measured with larger precision compared with the smaller studies. Inclusion of multiple surrogate endpoints may lead to a more substantial gain in precision in other disease areas and when data on some outcomes in some of the studies are missing at random 28. Deviance information criteria (DIC) obtained for the two trivariate models using the complete data, showed at the bottom of Table 4, suggests that the fit by both models is comparable. The model assuming conditional independence is simpler and easier to implement as shown in Section 5. It also requires fewer parameters to estimate as discussed further in Section 6. If such assumption can be justified, the model with the structured covariance may be more practical.

## 4 Simulation and sensitivity analysis

### 4.1 Methods

A simulation study was carried out to compare the performance of the three models: a bivariate model with a single surrogate endpoint and the two trivariate models with two surrogate endpoints, the model with unstructured between‐study covariance matrix and with the structured covariance matrix. Data were simulated under two scenarios considering alternative covariance structures: unstructured, by simulating data from the model (1)+(10) and structured by simulating data from the model (1)+(12). The parameters of the between‐study model were set to be comparable with those corresponding to the RRMS data, namely, *τ*_1,2,3_=0.5 and $\rho_{b}^{12,13,23}=0.8$ and *η*_1_=−0.3.

The regression coefficients and the conditional variances were obtained from the between‐study standard deviations and correlations following the formulae (11) and (13) for the models with unstructured and structured covariance matrices, respectively. The within‐study correlations $\rho_{\mathrm{wi}}^{\mathrm{jk}}$ were set to the same values as in the example in RRMS (obtained from the Prentice's criteria). The within‐study variances were simulated by drawing the precisions (inverse variances) from the gamma distributions; *σ*_1(2,3)*i*_=1/*P*_1(2,3)*i*_, *P*_1(2,3)*i*_∼*Γ*(*α*_1(2,3)_,*θ*_1(2,3)_), where *α*_1(2,3)_ are the shape parameters and *θ*_1(2,3)_ the scale parameters, which were obtained using the method of moments: *E*(*P*_1,2,3_) = *α*_1,2,3_/*ξ*_1,2,3_, $V(P_{1,2,3})=\alpha_{1,2,3}/\xi_{1,2,3}^{2}$, where *ξ*_1,2,3_=1/*θ*_1,2,3_ is a rate parameter. By summarising the inverse variances from the RRMS data, the following parameters were obtained: *E*(*P*_1_) = 30, *E*(*P*_2_) = 150, *E*(*P*_3_) = 25, *V*(*P*_1_) = 420, *V*(*P*_2_) = 15000 and *V*(*P*_2_) = 275, giving the following shape and rate parameters: *α*_1_=2.14, *α*_2_=1.5, *α*_3_=2.3, *ξ*_1_=0.07, *ξ*_2_=0.01 and *ξ*_3_=0.09. Because of the structure of the gamma distribution, some of the simulated precisions were very close to zero, resulting in very large variances. This led to some problems with the estimation. To overcome this issue, a constraint was placed on the simulated value of the precision by discarding the precisions resulting in variances larger than 2 (this number was taken as an arbitrary cut off, large enough to be much larger than the variances in the RRMS data and hence including all plausible variances in the population but small enough not to produce problems with the estimation). R code for the data simulation is included in the Web Supplement C.

The cross‐validation procedure is applied to each simulated data set. This time, however, the true effect *μ*_3*n*_ in a validation study *n* is known as it has been simulated, so the cross‐validation can be performed on the true effects (which in real circumstances we would like to predict) by comparing the simulated *μ*_3*n*_ with predicted interval of ${\hat{\mu }}_{3n}$ with the corresponding variance $\mathrm{var}({\hat{\mu }}_{3n}|Y_{1n},Y_{2n},\sigma_{1n},\sigma_{2n},Y_{1(-n)},Y_{2(-n)},Y_{3(-n)})$. The two trivariate models and also the bivariate model are applied to the simulated data sets to compare their performance, by estimating bias of the mean ${\hat{\mu }}_{3n}$, root‐mean‐square error (RMSE), coverage of 95% credible intervals (CrI) and the potential reduction of the width of the predicted interval by calculating the ratios of the width of the interval from a trivariate model *w*_3*d*_ (with two surrogate endpoints) with the width of the predicted interval from the bivariate model *w*_2*d*_.

### 4.2 Sensitivity analysis

To investigate the sensitivity of the methods to the assumption of normality of the data, another simulation study was carried out where the data were simulated from multivariate *t*‐distribution as well as mixture normal distribution.

#### 4.2.1 Simulation study with *t*‐distribution

To simulate data from the*t*‐distribution with unstructured covariance matrix, the true treatment effects on the three outcomes were generated from the following model:

(14) $$\left\{\begin{array}{l} \mu_{1i}∼\text{t}(\eta_{1},\nu_{1},\mathrm{df})\\ \mu_{2i}|\mu_{1i}∼\text{t}(\eta_{2i},\nu_{2},\mathrm{df})\\ \eta_{2i}=\lambda_{20}+\lambda_{21}\mu_{1i}\\ \mu_{3i}|\mu_{1i},\mu_{2i}∼\text{t}(\eta_{3i},\nu_{3},\mathrm{df})\\ \eta_{3i}=\lambda_{30}+\lambda_{31}\mu_{1i}+\lambda_{32}\mu_{2i}, \end{array}\right.$$

and from the *t*‐distribution with structured covariance matrix, from the following model

(15) $$\left\{\begin{array}{l} \mu_{1i}∼\text{t}(\eta_{1},\nu_{1},\mathrm{df})\\ \mu_{2i}|\mu_{1i}∼\text{t}(\eta_{2i},\nu_{2},\mathrm{df})\\ \eta_{2i}=\lambda_{20}+\lambda_{21}\mu_{1i}\\ \mu_{3i}|\mu_{1i},\mu_{2i}∼\text{t}(\eta_{3i},\nu_{3},\mathrm{df})\\ \eta_{3i}=\lambda_{30}+\lambda_{32}\mu_{2i}, \end{array}\right.$$

where $\nu_{1,2,3}=\left(\psi_{1,2,3}^{2}(\mathrm{df}-2)\right)/\mathrm{df}$ and *d* *f* = 4. The individual study estimates were simulated from the trivariate *t*‐distribution

(16) $$\left(\begin{array}{l} Y_{1i}\\ Y_{2i}\\ Y_{3i} \end{array}\right)∼\mathrm{MV}\text{t}\left(\left(\begin{array}{l} \mu_{1i}\\ \mu_{2i}\\ \mu_{3i} \end{array}\right),\Sigma_{i},\mathrm{df}\right),\;\Sigma_{i}=\left(\begin{array}{lll} \sigma_{1i}^{2} & \sigma_{1i}\sigma_{2i}\rho_{\mathrm{wi}}^{12} & \sigma_{1i}\sigma_{3i}\rho_{\mathrm{wi}}^{13}\\ \sigma_{2i}\sigma_{1i}\rho_{\mathrm{wi}}^{12} & \sigma_{2i}^{2} & \sigma_{2i}\sigma_{3i}\rho_{\mathrm{wi}}^{23}\\ \sigma_{3i}\sigma_{1i}\rho_{\mathrm{wi}}^{13} & \sigma_{3i}\sigma_{2i}\rho_{\mathrm{wi}}^{23} & \sigma_{3i}^{2} \end{array}\right).$$

The simulation was conducted in R software using the rt command (and scaling the simulated data by $\psi_{1,2,3}\sqrt{\left((\mathrm{df}-2)/\mathrm{df}\right)}+\eta_{1}$) for univariate*t*‐distributions in the between‐study models (14) and (15) and the rmvt in the within‐study model (16).

#### 4.2.2 Simulation study with mixture normal distribution

Data with more severe departure from normality were generated by the use of mixture normal distributions. This was achieved by replacing the univariate normal distribution for the true treatment effect on the first outcome in the models (10) and (12) with the mixture normal distribution

(17) $$\mu_{1i}∼p_{1}*N\left(\eta_{1},\psi_{1}^{2}\right)+p_{2}*N\left(\eta_{1}-4*\psi_{1},\psi_{1}^{2}\right)+p_{3}*N\left(\eta_{1}+4*\psi_{1},\psi_{1}^{2}\right),$$

with *p*_1_=0.5, *p*_2_=0.3 and *p*_3_=0.2. This deviation from normality feeds through to the true effects *μ*_2*i*_ and *μ*_3*i*_ by the linear association of those effects with *μ*_1*i*_. These now non‐normal true effects *μ*_1(2,3)*i*_ are then used as mean values when generating the within study data from the multivariate normal distribution giving data with ‘distorted’ normality.

### 4.3 Results

Data sets including the treatments effects (and corresponding sampling variances) on three outcomes in 15 studies were generated in 1000 simulations for each scenario. 0.1% of simulation runs were discarded because of precisions resulting in too high variances, as explained in the methods Section 4.1. Simulation results are presented in Table 5. The bias of mean predicted effect ${\hat{\mu }}_{3n}$ for a validation study *n* was comparable across all models and data scenarios. The RMSE was larger when data were simulated from a model with unstructured covariance matrix, regardless of the distribution or model fitted to the data. All models (the bivariate and the two trivariate) seemed to perform equally well, giving coverage of 95% credible interval close to 95% for most scenarios, except for the data generated from the mixture normal distribution where the coverage was slightly inflated to 98% (because of the three normal distributions being approximated by models with a single normal distribution, leading to the inflated variance of predictions). For data generated from either multivariate normal or *t*‐distribution with unstructured covariance matrix, both trivariate models gave on average 4% reduction of the width of the predicted interval compared with the intervals obtained from the bivariate model. When data was simulated using normal or *t*‐distribution with structured covariance matrix, the trivariate model with unstructured covariance gave on average 2% reduction in uncertainty around the predicted effect compared with the uncertainty around predictions obtained from the bivariate model, while the model with structured covariance matrix gave predictions with intervals 5% narrower compared with those obtained from the model with a single surrogate endpoint. When data were simulated from the mixture of normal distributions, the trivariate model with unstructured covariance did not produce any gain in precision of predictions, while the model with structured covariance matrix gave predicted intervals that were on average 9% narrower compared with those obtained from the bivariate model.

**Table 5:** Results of simulation studies.

Meta‐analysis model	Bias of mean ${\hat{\mu }}_{3n}$	RMSE of ${\hat{\mu }}_{3n}$	Coverage of 95% CrI for ${\hat{\mu }}_{3n}$	Median *w*_3*d*_/*w*_2*d*_
Scenario 1: Data simulated from normal TRMA with UCM				
BRMA	−0.002	0.46	0.96	
TRMA UCM	0.003	0.45	0.96	0.96
TRMA SCM	−0.001	0.46	0.95	0.96
Scenario 2: Data simulated from normal TRMA with SCM				
BRMA	0.006	0.36	0.95	
TRMA UCM	0.003	0.36	0.95	0.98
TRMA SCM	0.006	0.35	0.94	0.95
Scenario 3: Data simulated from TRMA with UCM and *t*‐distribution				
BRMA	−0.002	0.48	0.95	
TRMA UCM	−0.004	0.47	0.95	0.96
TRMA SCM	−0.002	0.48	0.94	0.96
Scenario 4: Data simulated from TRMA with SCM and *t*‐distribution				
BRMA	−0.0001	0.36	0.95	
TRMA UCM	0.002	0.37	0.95	0.98
TRMA SCM	0.0002	0.36	0.94	0.95
Scenario 5: Data simulated from TRMA with UCM and mixture normal				
BRMA	−0.007	0.49	0.98	
TRMA UCM	0.003	0.47	0.98	1.00
TRMA SCM	−0.001	0.47	0.97	0.91
Scenario 6: Data simulated from TRMA with SCM and mixture normal				
BRMA	−0.002	0.36	0.98	
TRMA UCM	0.0001	0.37	0.98	1.01
TRMA SCM	−0.0001	0.36	0.97	0.91

RMSE, root‐mean‐squared‐error; CrI, credible interval

UCM, unstructured covariance matrix; SCM, structured covariance matrix; TRMA, trivariate random effects meta‐analysis;

*w*_3*d*_ (*w*_2*d*_), width of the predicted interval from TRMA (BRMA)

MC errors of the predicted mean effects were less than 0.012 in scenarios 1–4 and less than 0.015 and 0.025 in scenarios 5 and 6, respectively

### 4.4 Discussion of the results

The outcomes of the simulation study were broadly in agreement with those obtained from the case study. The predicted intervals obtained from TRMA models were narrower compared with those obtained from BRMA, but this reduction was less pronounced when using the TRMA UCM model on the data simulated from a model with structured covariance matrix (one of the surrogates correlated to the other surrogate but less so to the final outcome; scenarios 2, 4 and 6 in Table 5 and also in the RRMS case study). Using TRMA UCM on data simulated from the same model gives the same reduction in uncertainty as when using TRMA SCM but this effect of the addition of the second surrogate on the uncertainty around the predicted effects diminished with the departure from normality (scenario 5). There was no effect of adding the second surrogate endpoint on the RMSE which was almost the same across the three methods within each scenario. When data were simulated from non‐normal distribution (*t*‐distribution or mixture normal) with structured covariance matrix, the RMSE was slightly larger when using TRMA UCM (compared with BRMA or TRMA SCM). This is likely due to the TRMA UCM model forcing too rigid correlation structure on the data leading to bias when making predictions for outlying observations in data with not as strong correlation pattern. When data represents all outcomes correlated (from a distribution with unstructured covariance matrix), both TRMA models seem to perform equally well, with slightly smaller RMSE when using TRMA UCM if the data are normally distributed. However, for non‐normally distributed data, gain in precision is only present when using TRMA SCM. When data corresponds to the scenario with the structured covariance matrix the TRMA SCM model seems to perform better than TRMA UCM in terms of both RMSE and the uncertainty of the predictions.

## 5 Multivariate random effects meta‐analysis

Methodology introduced in Section 2 can be extended to a scenario with multiple surrogate endpoints. Suppose, we have estimates of treatment effects observed on *N* outcomes, $Y_{1i},Y_{2i},\ldots ,Y_{\mathrm{Ni}}$ in each study *i*, and *Y*_*N*_ is the final clinical outcome of interest, while $Y_{1},\ldots ,Y_{N-1}$ are intermediate surrogate endpoints. If the estimates of the treatment effects on all of the outcomes are assumed normally distributed and correlated, then they follow a multivariate normal distribution:

(18) $$\left(\begin{array}{l} Y_{1i}\\ Y_{2i}\\ \vdots \\ Y_{\mathrm{Ni}} \end{array}\right)∼N\left(\left(\begin{array}{l} \mu_{1i}\\ \mu_{2i}\\ \vdots \\ \mu_{\mathrm{Ni}} \end{array}\right),\Sigma_{i}\right),\;\Sigma_{i}=\left(\begin{array}{llll} \sigma_{1i}^{2} & \sigma_{1i}\sigma_{2i}\rho_{\mathrm{wi}}^{12} & \cdots & \sigma_{1i}\sigma_{\mathrm{Ni}}\rho_{\mathrm{wi}}^{1N}\\ \sigma_{2i}\sigma_{1i}\rho_{\mathrm{wi}}^{12} & \sigma_{2i}^{2} & \cdots & \sigma_{2i}\sigma_{\mathrm{Ni}}\rho_{\mathrm{wi}}^{2N}\\ \vdots & \vdots & \ddots & \vdots \\ \sigma_{\mathrm{Ni}}\sigma_{1i}\rho_{\mathrm{wi}}^{1N} & \sigma_{\mathrm{Ni}}\sigma_{2i}\rho_{\mathrm{wi}}^{2N} & \cdots & \sigma_{\mathrm{Ni}}^{2} \end{array}\right)$$

In the aforementioned model, outcomes *Y*_1*i*_, *Y*_2*i*_, … and *Y*_*Ni*_ are assumed to be estimates of correlated true effects *μ*_1*i*_, *μ*_2*i*_, … and *μ*_*Ni*_ with corresponding within‐study covariance matrices *Σ*_*i*_ of the estimates. These study‐level effects follow a multivariate normal distribution with means $\left(\beta_{1},\beta_{2},\ldots ,\beta_{N}\right)$ and covariance **T**,

(19) $$\left(\begin{array}{l} \mu_{1i}\\ \mu_{2i}\\ \vdots \\ \mu_{\mathrm{Ni}} \end{array}\right)∼N\left(\left(\begin{array}{l} \beta_{1}\\ \beta_{2}\\ \vdots \\ \beta_{N} \end{array}\right),T\right),\;T=\left(\begin{array}{llll} \tau_{1}^{2} & \tau_{1}\tau_{2}\rho_{b}^{12} & \cdots & \tau_{1}\tau_{N}\rho_{b}^{1N}\\ \tau_{2}\tau_{1}\rho_{b}^{12} & \tau_{2}^{2} & \cdots & \tau_{2}\tau_{N}\rho_{b}^{2N}\\ \vdots & \vdots & \ddots & \vdots \\ \tau_{N}\tau_{1}\rho_{b}^{1N} & \tau_{N}\tau_{2}\rho_{b}^{2N} & \cdots & \tau_{N}^{2} \end{array}\right).$$

In this hierarchical framework, Equations (18) and (19) describe the within‐study and the between‐study models, respectively. The between‐study model can be reparameterised to extend the scenarios for modelling of surrogate endpoints described in Sections 2.3, 2.4 and 2.5 to the multivariate case, using the unstructured and structured covariance matrices.

### 5.1 Product normal formulation with unstructured covariance matrix

Assuming that true treatment effects on all the outcomes are correlated, we can parameterise the between‐study model (19) in the form of product normal formulation by extending model (10) to

(20) $$\left\{\begin{array}{l} \mu_{1i}∼N\left(\eta_{1},\psi_{1}^{2}\right)\\ \mu_{2i}|\mu_{1i}∼N\left(\eta_{2i},\psi_{2}^{2}\right)\\ \eta_{2i}=\lambda_{20}+\lambda_{21}\mu_{1i}\\ \mu_{3i}|\mu_{1i},\mu_{2i}∼N\left(\eta_{3i},\psi_{3}^{2}\right)\\ \eta_{3i}=\lambda_{30}+\lambda_{31}\mu_{1i}+\lambda_{32}\mu_{2i}\\ \vdots \\ \mu_{\mathrm{Ni}}|\mu_{1i},...,\mu_{(N-1)i}∼N\left(\eta_{\mathrm{Ni}},\psi_{N}^{2}\right)\\ \eta_{\mathrm{Ni}}=\lambda_{N0}+\lambda_{N1}\mu_{1i}+\cdots +\lambda_{N(N-1)}\mu_{(N-1)i}. \end{array}\right.$$

In this model, prior distributions need to be placed on all the parameters. Non‐informative normal distributions are placed on the mean effect *η*_1_∼*N*(0,1000) and the intercepts $\lambda_{20},\ldots ,\lambda_{N0}∼N(0,1000)$. Similarly as in the trivariate case, we place prior distributions on the between‐study correlations and between‐study standard deviations (elements of matrix *T* in (19) for which we are more likely to anticipate a range of values or, in some applications can obtain an external information to construct informative prior distributions for them). The relationships between the model hyper‐parameters (conditional variances $\psi_{1}^{2},\psi_{2}^{2},\ldots ,\psi_{N}^{2}$ and slopes $\lambda_{21},\lambda_{31},\lambda_{32},\ldots ,\lambda_{N1},\lambda_{N2},\ldots ,\lambda_{N(N-1)}$) and the between‐study parameters (correlations and standard deviations) give implied prior distributions for those hyper‐parameters and also ensure that the between‐study covariance is positively defined,

(21) $$\left\{\begin{array}{l} \psi_{1}^{2}=\tau_{1}^{2}\\ \psi_{2}^{2}=\tau_{2}^{2}-\lambda_{21}^{2}\tau_{1}^{2}\\ \psi_{3}^{2}=\tau_{3}^{2}-\lambda_{31}^{2}\tau_{1}^{2}-\lambda_{32}^{2}\tau_{2}^{2}\\ \vdots \\ \psi_{N}^{2}=\tau_{N}^{2}-\lambda_{N1}^{2}\tau_{1}^{2}-\lambda_{N2}^{2}\tau_{2}^{2}-\cdots -\lambda_{N(N-1)}^{2}\tau_{N-1}^{2}. \end{array}\right.$$

(22) $$\left\{\begin{array}{l} \lambda_{21}=\rho_{b}^{12}\tau_{2}/\tau_{1}\\ \lambda_{31}=\rho_{b}^{13}\tau_{3}/\tau_{1}-\lambda_{21}\\ \lambda_{32}=\left(\rho_{b}^{23}\tau_{2}\tau_{3}-\lambda_{21}\lambda_{31}\tau_{1}^{2}\right)/\tau_{2}^{2}\\ \vdots \\ \lambda_{\mathrm{NQ}}=\left(\rho_{b}^{\mathrm{QN}}\tau_{Q}\tau_{N}-\overset{N-1}{\underset{P=1,P\neq Q}{\sum }}\lambda_{\mathrm{NQ}}\mathrm{Cov}(\mu_{P},\mu_{Q})\right)/\tau_{Q}^{2} \end{array}\right.$$

These relationships are obtained by calculating the variances and correlations in terms of the hyperparameters and rearranging the equations for the variances and solving the set of simultaneous equations for correlations. Details are given in Appendix B.1. This becomes a complex task in higher dimensions. Alternative and simpler model can be used by assuming a structure of the between‐study covariance matrix as described in Section 5.2.

#### 5.1.1 Criteria for surrogate markers

In the case of multiple endpoints with *N* − 1 surrogate markers, the effects of treatment on all biomarkers may jointly mediate the treatment effect on the target outcome. For the combined effect on the biomarkers to fully mediate the effect on the target outcome, we expect the intercept *λ*_*N*0_=0 and the conditional variance $\psi_{N}^{2}=0$. The association between the effect on target outcome and each of the surrogate endpoints is expected not to be zero; *λ*_*NX*_≠0, ( X=1,…,N−1). Similarly, the relationship between effects on other outcomes can be investigated. For example, if we consider outcomes *Y*_1_ to *Y*_*M* − 1_ to be potential surrogates to outcome *Y*_*M*_ (*M* < *N*), then coefficients for *η*_*Mi*_, namely, $\lambda_{M0},\lambda_{M1},\ldots ,\lambda_{M(M-1)}$ and $\psi_{M}^{2}$, are investigated in the same manner, giving an option for considering multiple ‘final’ endpoints at the same time.

### 5.2 Product normal formulation with structured covariance matrix

Scenario with two surrogate endpoints and final outcome measured ‘in sequence’, described in Section 2.5 can be extended to the multivariate case. If we imagine that the *N* outcomes are ordered in a sequence (for example according to measurement time or other reasons that would impose such correlation structure), a conditional independence between any pair of outcomes that are not ‘neighbours’ can be assumed, conditional on the outcomes placed in the sequence in between that particular pair.

This leads to a structure being placed on the between‐study covariance matrix in such a way to fully take into account of the correlations between the treatment effect on pairs of outcomes (for example those that are measured one after another in a time sequence, but assume conditional independence of other effects). The elements of the precision matrix *T* ^−1^ corresponding to the effects that are conditionally independent become zero and only those on diagonal and immediate off‐diagonals are non‐zero. The between‐study model (19) is then parameterised in the product normal,

(23) $$\left\{\begin{array}{l} \mu_{1i}∼N\left(\eta_{1},\psi_{1}^{2}\right)\\ \mu_{2i}|\mu_{1i}∼N\left(\eta_{2i},\psi_{2}^{2}\right)\\ \eta_{2i}=\lambda_{20}+\lambda_{21}\mu_{1i}\\ \mu_{3i}|\mu_{2i}∼N\left(\eta_{3i},\psi_{3}^{2}\right)\\ \eta_{3i}=\lambda_{30}+\lambda_{32}\mu_{2i}\\ \vdots \\ \mu_{\mathrm{Ni}}|\mu_{(N-1)i}∼N\left(\eta_{\mathrm{Ni}},\psi_{N}^{2}\right)\\ \eta_{\mathrm{Ni}}=\lambda_{N0}+\lambda_{N(N-1)}\mu_{(N-1)i}, \end{array}\right.$$

Similarly as in previous models (the trivariate and the multivariate with unstructured between‐study covariance), the parameters of the above model can be expressed in terms of the elements of the between‐study covariance matrix **T** (19):

(24) $$\psi_{1}^{2}=\tau_{1}^{2},\;\;\;\psi_{2}^{2}=\tau_{2}^{2}-\lambda_{21}^{2}\tau_{1}^{2},\;\;\;\psi_{3}^{2}=\tau_{3}^{2}-\lambda_{32}^{2}\tau_{2}^{2},\;\;\ldots ,\;\;\psi_{N}^{2}=\tau_{N}^{2}-\lambda_{N(N-1)}^{2}\tau_{N-1}^{2}$$

and

(25) $$\left\{\begin{array}{l} \lambda_{21}=\rho_{b}^{12}\frac{\tau_{2}}{\tau_{1}}\\ \lambda_{32}=\rho_{b}^{23}\frac{\tau_{3}}{\tau_{2}}\\ \vdots \\ \lambda_{N(N-1)}=\rho_{b}^{(N-1)N}\frac{\tau_{N}}{\tau_{\left(N-1\right)}} \end{array}\right.$$

obtained from the formulae for the correlations in Appendix B.2. Non‐informative prior distributions are then placed on the between‐study correlations and standard deviations, $\rho_{b}^{12},\rho_{b}^{23},\rho_{b}^{34},\ldots ,\rho_{b}^{(N-1)N}∼\mathrm{dunif}(-1,1)$, $\tau_{1},\ldots ,\tau_{N}∼N(0,10)I(0,)$, as well as the remaining, independent parameters, *η*_1_∼*N*(0,1000), $\lambda_{20},\ldots ,\lambda_{N0}∼N(0,1000)$. In this form, the model is much easier to implement compared with the model with the unstructured covariance matrix in Section 5.1.

## 6 Discussion

**Figure 4:**
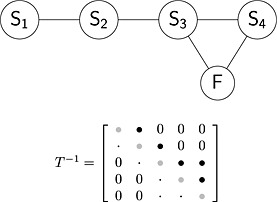
Example of a scenario of modelling multiple surrogate endpoints with a choice of a correlation structure.

We have developed a multivariate meta‐analytic framework to include multiple surrogate endpoints when predicting the treatment effect on the final clinical outcome in an early drug evaluation process. The validation process discussed here aims to evaluate how well the effect of a treatment on multiple surrogate endpoints can jointly predict treatment effect on the final clinical outcome. Two approaches were developed, one assuming all effects being correlated giving an unstructured between‐study covariance matrix and a model assuming conditional independence between the effects on some of the endpoints giving a structured covariance matrix. The first model makes fewer assumptions about the data but requires estimation of a large number of parameters (which may be difficult if the number of studies is relatively low) and also may be difficult to implement in higher dimensions. The second model makes an assumption of conditional independence of some effects but leads to a reduced number of parameters to estimate and is easier to implement as the relationships between the parameters of the model and the elements of the between‐study covariance matrix have a simple form. For example, in a scenario with five endpoints (four surrogate endpoints and one final outcome), the model with unstructured covariance matrix is set up to estimate five between study variances and ten between‐study correlations, while the model assuming conditional independence with structured between‐study covariance matrix estimates only four between‐study correlations. However the assumption of conditional independence of some of the outcomes may lead to underestimating some correlations which may impact on predictions. This modelling framework, however, allows for flexible modelling of the correlation structure where a choice can be made about which correlations need to be fully taken into account. A model ‘in between’ those two discussed can be implemented, such as the one showed schematically in Figure 4 which extends the scenario with five endpoints in sequence (structured covariance matrix model) by taking into account of the correlation between the final and the second to last outcome (now removing the zero from the element [3,5] of the precision matrix *T* ^−1^ which was present in the sequential model with conditional independence of effects on outcomes three and five). The desirable correlation is taken into account, while an assumption of conditional independence of the remaining outcomes is still made thereby reducing the number of correlations from ten in the model with unstructured covariance matrix to only five. See all three scenarios represented graphically in the Web Supplement D. The results of the case study and the simulation study suggest that the choice of the model (in terms of the covariance structure) when made in line with the correlation structure of the data will lead to better predictions.

Further research is required to extend the aforementioned methodology to more exact methods for Binomial or count data to relax the assumption of normality, for example, by adopting methods similar to those introduced by Stijnen *et al*. 29 using exact likelihood, by means of a generalised linear mixed models. However, our simulation study showed robustness of the methods to the departures from normality of the data. Another limitation is the prediction of the treatment effect ‘estimate’ rather than the true effect. Ideally, we want to know how our predicted treatment effect compares with the true treatment effect. However, although the models have the ability to predict the true effects, such effect cannot be used for validation based on the real data as the true treatment effect is unknown which limits the cross‐validation to comparing the predicted treatment effect ‘estimates’ with the observed (but pretended missing) treatment effects for the final outcome.

The approach to validation of surrogate endpoints presented here focusses mainly on validating the endpoints as good (joint) predictors of the clinical benefit on the final outcome. It is now well established that the surrogacy has to be validated on both individual and study level. Prentice's criteria can be used to validate surrogate endpoints on the individual level when IPD are available. The use of the Prentice's criteria to estimate the within‐study correlations between the treatment effects on different outcomes (by assuming one endpoint in each pair of outcomes can be considered a surrogate to the other) provides a bridge between validation on the two levels. This approach may be useful when the sharing of patient data from multiple clinical trials is problematic, but owners of the data may be willing to share Prentice's criteria.

There are some limitations of Prentice's criteria for surrogacy. As discussed by a number of authors 30, 31, 32, satisfying those criteria does not guarantee a causal relationship between the treatment effects on the surrogate and the final outcome. An example of data scenario where Prentice's fourth criterion is satisfied but it does not support a claim of causality can be found in Buyse *et al*, who discuss a number of approached to evaluation of surrogate endpoints 32. As pointed out by Joffe and Greene 30, the statistical model describing the Prentice's criteria does not account for the common causes of the surrogate and the final outcomes. The fourth Prentice's criteria, sometimes referred to as the surrogacy criteria, is based on the assumption that the treatment effect can be partitioned into direct and indirect effects with the indirect effect being the part mediated by the surrogate and the direct being the part of the effect not mediated by the surrogate. Ignoring the common causes by modelling the direct effect on the final outcome by conditioning on the effect on the surrogate is, however, not equivalent with experimentally controlling the surrogate, as pointed out by Pearl 33. As discussed by VanderWeele 31, this can lead to the ‘surrogate paradox’ where despite the positive association between treatment and the surrogate and the surrogate and the final outcome, the association between the treatment and the final outcome may be negative. Chen *et al* 34 referred to this phenomena as effect reversal.

This issue of the causal effect mostly affects surrogate evaluation when such analysis is based on a single study. The meta‐analytic approach, such as described in our paper, follows the causal association paradigm which, as discussed by Joffe and Greene, is based on establishing the association between the treatment effects on the candidate surrogate endpoint and on the final outcome (rather than modelling the effect of surrogate on the final outcome) 30. The authors point out that this approach is more useful for evaluation of surrogate endpoints as it is free from the restrictions of the causal effect paradigm as the causal association does not require experimental manipulation of the surrogate. The meta‐analytic approach to the causal association study involves associations between quantities that derive directly from randomisation and as such are average causal effects. Meta‐analytic approach, such as proposed in our study, is based on data from a number of studies or subgroups and is likely to include heterogeneous treatment contrasts which is an obvious advantage over evaluation based on a single study. Such a single trial validation cannot guarantee that an association between effects confirmed based on individual data under one treatment will hold in other interventions. Moreover, Alonso *et al*, who investigated the relationship between the causal inference and meta‐analytic approaches to the surrogate endpoint evaluation, have shown that causal effect of the surrogate on the final outcome validated based on a single study may not be confirmed in the meta‐analytic setting, in particular, when the between‐study heterogeneity is large and the causal effect is weak 35. The authors have also concluded that a surrogate endpoint that successfully validated in a meta‐analytic setting, on both individual and trial level, is likely to be confirmed when evaluated based on causal inference framework.

In this paper, we used the Prentice's criteria to estimate the within‐study correlation between the treatment effects, as ignoring the within‐study correlation has known consequences 15. Moreover, ideally, the correlation should be obtained for each study independently to account for potential differences in the association depending on the treatment contrast. The main goal in the development of these models was building a framework under which all available evidence can be used to predict future treatment effect on the final outcome while taking into account of uncertainty around relevant parameters. In particular, these approaches have advantage over simple regression models as they take into account measurement error of the treatment effect on surrogate endpoints ignoring which can impact on predictions 12. Further research on putting this work in the framework of causal mediation could provide solution to some of the aforementioned limitations.

## Supporting information

supporting Info Item

## Appendix A Within‐study model for relapsing remitting multiple sclerosis

### A.1

In this Section, the details of how the data in Tables 1 and 2 are used to obtain the within‐study standard variances and correlations.

#### A.1 Within‐study variances

The MRI effect is modelled on the log rate ratio (RR) scale,

(A1) $$Y_{1i}=\log\mathrm{MRIRR}=\log(Rm_{E}/Rm_{C})$$

with

(A2) $$\sigma_{1i}^{2}=\mathrm{Var}(\log\mathrm{MRIRR})=\mathrm{Var}(\log Rm_{E}-\log Rm_{C})=\mathrm{Var}(\log Rm_{E})+\mathrm{Var}(\log Rm_{C}),$$

where subscript *C* and *E* stand for the control and experimental arms, respectively. From the delta method

(A3) $$\mathrm{Var}(\log Rm_{X})=\frac{1}{{\left(Rm_{X}\right)}^{2}}\mathrm{Var}(Rm_{X})={\left(\frac{\mathrm{SE}(Rm_{X})}{Rm_{X}}\right)}^{2}$$

in each arm *X*.

The relative treatment effect on relapses is modelled by the log annualised relapse rate ratio (ARRR) scale,

(A4) $$Y_{2i}=\log\mathrm{ARRR}=\log\left(\frac{\mathrm{AR}R_{E}}{\mathrm{AR}R_{C}}\right)$$

with

(A5) $$\sigma_{2i}^{2}=\mathrm{Var}(\log\mathrm{ARRR})=\mathrm{Var}(\log\mathrm{AR}R_{E}-\log\mathrm{AR}R_{C})=\mathrm{Var}(\log\mathrm{AR}R_{E})+\mathrm{Var}(\log\mathrm{AR}R_{C}),$$

where *A* *R* *R*_*X*_=*A* *R* *r*_*X*_/*N* *r*_*X*_ is the annualised relapse rate in arm X. From delta method

(A6) $$\begin{array}{ccc} \mathrm{Var}(\log\mathrm{AR}R_{X}) & = & \mathrm{Var}(\log(\mathrm{RelRat}e_{X}/\mathrm{Ft}))=\mathrm{Var}(\log(\mathrm{RelRat}e_{X})-\log(\mathrm{Ft}))\\ = & \mathrm{Var}(\log(\mathrm{RelRat}e_{X}))\\ = & \frac{1}{{\left(\mathrm{mean}(\mathrm{RelRat}e_{X})\right)}^{2}}\mathrm{Var}(\mathrm{RelRat}e_{X})\\ = & \frac{1}{\mathrm{mean}(\mathrm{RelRat}e_{X})}=\frac{12}{\mathrm{AR}r_{x}\times \mathrm{Ft}}, \end{array}$$

where *F* *t* is the follow‐up time in months and *R* *e* *l* *R* *a* *t* *e*_*X*_ is relapse rate in arm X.

The relative treatment effect on disability progression is modelled on the log odds ratio (OR) scale,

(A7) $$Y_{3i}=\log\mathrm{OR}=\log\left(\frac{Rd_{E}(Nd_{C}-Rd_{C})}{Rd_{C}(Nd_{E}-Rd_{E})}\right)$$

with the corresponding variance

(A8) $$\sigma_{3i}^{2}=\mathrm{Var}(\log\mathrm{OR})=\frac{1}{Rd_{E}}+\frac{1}{Nd_{E}-Rd_{E}}+\frac{1}{Rd_{C}}+\frac{1}{Nd_{C}-Rd_{C}}.$$

The within‐study correlations $\rho_{\mathrm{wi}}^{\mathrm{jk}}$ are required to complete the within‐study model. Because the correlations are not available for any of the studies and the IPD are not available for any of the studies, but the Prentice's criteria are available for one of the studies (Li for combined cohorts C and D), the approach derived in Section 2.2.2 is adopted here to calculate the within‐study correlations for this particular study which is then assumed to be the same in all of the studies in meta‐analysis.

#### A.2 Within‐study correlations

Let *ξ* correspond to the index of the study by Li *et al*.

##### A.0.2.1 The within‐study correlation between the estimates of the treatment effects on MRI and on the relapse rate

In this study,

$$\rho_{\mathrm{w\xi}}^{12}=\Sigma_{\xi }[1,2]/\sqrt{\sigma_{1\xi }^{2}\sigma_{2\xi }^{2}}$$

and the covariance between log MRI RR and log ARRR, *Σ*_*ξ*_[1,2] can be expressed as

(A9) $$\begin{array}{ccc} \Sigma_{\xi }[1,2]=\Sigma_{\xi }[2,1] & = & \mathrm{Cov}(\log\mathrm{ARRR},\log\mathrm{MRIRR})\\ = & \mathrm{Cov}(\log\mathrm{AR}R_{E}-\log\mathrm{AR}R_{C},\log Rm_{E}-\log Rm_{C})\\ = & \mathrm{Cov}(\log\mathrm{AR}R_{E},\log Rm_{E})+\mathrm{Cov}(\log\mathrm{AR}R_{C},\log Rm_{C}) \end{array}$$

and

(A10) $$\begin{array}{ccc} \mathrm{Cov}(\log\mathrm{AR}R_{X},\log Rm_{X}) & = & \mathrm{Cov}(\log\mathrm{AR}r_{X}-\log Nr_{X},\log Rm_{X})\\ = & \mathrm{Cov}(\log\mathrm{AR}r_{X},\log\mathrm{MR}m_{X})-\mathrm{Cov}(\log Nr_{X},\log Rm_{X})\\ = & \sqrt{\mathrm{Var}(\log\mathrm{AR}r_{X})\mathrm{Var}(\log Rm_{X})}\rho_{\mathrm{MR}}^{*}\\ -\sqrt{\mathrm{Var}(\log Nr_{X})\mathrm{Var}(\log Rm_{X})}{\hat{\rho }}_{\mathrm{MR}}\\ = & \sqrt{\mathrm{Var}(\log\mathrm{AR}r_{X})\mathrm{Var}(\log Rm_{X})}\rho_{\mathrm{MR}}^{*}. \end{array}$$

where the correlation $\rho_{\mathrm{MR}}^{*}$, as discussed in Section 2, equals to the adjusted association obtained using the formula (8)

(A11) $$\rho_{\mathrm{MR}}^{*}=\frac{\beta_{3}-\beta_{S3}}{\alpha_{3}}\sqrt{\omega_{\mathrm{\xi MM}}/\omega_{\mathrm{\xi RR}}}.$$

Prentice's criteria for the association between the log MRI rate and log relapse rate(*α*_3_, *β*_3_ and *β*_*S*3_ reported in Table 2) were obtained by fitting the model (6) to the full cohort of the study by Li *et al*. (rather than separately to the treatment arms). We assume that the correlation is the same in the treatment arms and the whole cohort and hence, the variances *ω*_*ξ**M**M*_=*V* *a* *r*(log*R* *m*_*E*_) + *V* *a* *r*(log*R* *m*_*C*_) and *ω*_*ξ**R**R*_=*V* *a* *r*(log*A* *R* *r*_*E*_) + *V* *a* *r*(log*A* *R* *r*_*C*_).

##### A.2.2 The within‐study correlation between the estimates of the treatment effects on relapse rate and on the disability progression

In this study the correlation can be obtained in a similar manner:

$$\rho_{\mathrm{w\xi}}^{23}=\Sigma_{\xi }[2,3]/\sqrt{\sigma_{2\xi }^{2}\sigma_{3\xi }^{2}}$$

and the covariance between log ARRR and log OR, *Σ*_*ξ*_[2,3], can be expressed as

(A12) $$\begin{array}{cc} \Sigma_{\xi }[2,3]=\Sigma_{\xi }[3,2] & =\mathrm{Cov}(\log\mathrm{ARRR},\log\mathrm{OR})\\ =\mathrm{Cov}(\log\mathrm{AR}R_{E}-\log\mathrm{AR}R_{C},\log OP_{E}-\log OP_{C})\\ =\mathrm{Cov}(\log\mathrm{AR}R_{E},\log OP_{E})+\mathrm{Cov}(\log\mathrm{AR}R_{C},\log OP_{C}) \end{array}$$

and

(A13) $$\begin{array}{cc} \mathrm{Cov}(\mathrm{logAR}R_{X},\mathrm{logO}P_{X}) & =\mathrm{Cov}(\log\mathrm{AR}r_{X}-\log Nr_{X},\log OP_{X})\\ =\mathrm{Cov}(\log\mathrm{AR}r_{X},\log OP_{X})-\mathrm{Cov}(\log Nr_{X},\log OP_{X})\\ =\sqrt{\mathrm{Var}(\log\mathrm{AR}r_{X})\mathrm{Var}(\log OP_{X})}\rho_{\mathrm{RP}}^{*}\\ -\sqrt{\mathrm{Var}(\log Nr_{X})\mathrm{Var}(\log OP_{X})}{\hat{\rho }}_{\mathrm{RP}}\\ =\sqrt{\mathrm{Var}(\log\mathrm{AR}r_{X})\mathrm{Var}(\log OP_{X})}\rho_{\mathrm{RP}}^{*} \end{array}$$

where *O* *P*_*X*_ refers to the odds of progression in arm *X* and $\mathrm{Var}(\mathrm{logO}P_{X})=\frac{1}{Rd_{X}}+\frac{1}{Nd_{X}-Rd_{X}}$. The correlation $\rho_{\mathrm{RP}}^{*}$ is the adjusted association obtained using the formula (8)

(A14) $$\rho_{\mathrm{RP}}^{*}=\frac{\beta_{2}-\beta_{S2}}{\alpha_{2}}\sqrt{\omega_{\mathrm{\xi RR}}/\omega_{\mathrm{\xi PP}}}.$$

Prentice's criteria for the association between the log relapse rate and log odds of disability (*α*_2_, *β*_2_ and *β*_*S*2_ reported in Table 2) were obtained by fitting the model (6) to the full cohort of the study by Li *et al*. hence, the variances *ω*_*ξ**R**R*_=*V* *a* *r*(log*A* *R* *R*_*E*_) + *V* *a* *r*(log*A* *R* *R*_*C*_) and *ω*_*ξ**P**P*_=*V* *a* *r*(log*O* *P*_*E*_) + *V* *a* *r*(log*O* *P*_*C*_).

##### A.2.3 The within‐study correlation between the estimates of the treatment effects on MRI and on the disability progression

In this study the correlation is obtained using similar approach:

$$\rho_{\mathrm{w\xi}}^{13}=\Sigma_{\xi }[1,3]/\sqrt{\sigma_{1\xi }^{2}\sigma_{3\xi }^{2}}$$

and the covariance between log MRI RR and log OR, *Σ*_*ξ*_[1,3] can be expressed as

(A15) $$\begin{array}{ccc} \Sigma_{i}[1,3]=\Sigma_{i}[3,1] & = & \mathrm{Cov}(\log\mathrm{MRIRR},\log\mathrm{OR})\\ = & \mathrm{Cov}(\log Rm_{E}-\log Rm_{C},\log OP_{E}-\log OP_{C})\\ = & \mathrm{Cov}(\log Rm_{E},\log OP_{E})+\mathrm{Cov}(\log Rm_{C},\log OP_{C}) \end{array}$$

and

(A16) $$\mathrm{Cov}(\log Rm_{X},\log OP_{X})=\sqrt{\mathrm{Var}(\log Rm_{X})\mathrm{Var}(\log OP_{X})}\rho_{\mathrm{MP}}^{*},$$

where

(A17) $$\rho_{\mathrm{MP}}^{*}=\frac{\beta_{1}-\beta_{S1}}{\alpha_{1}}\sqrt{\omega_{\mathrm{\xi MM}}/\omega_{\mathrm{\xi PP}}}.$$

Prentice's criteria for the association between the log MRI rate and log odds of disability (*α*_1_, *β*_1_ and *β*_*S*1_ reported in Table 2) were obtained by fitting the model (6) to the full cohort of the study by Li *et al*. hence, the variances *ω*_*ξ**M**M*_=*V* *a* *r*(log*R* *m*_*E*_) + *V* *a* *r*(log*R* *m*_*C*_) and *ω*_*ξ**P**P*_=*V* *a* *r*(log*O* *P*_*E*_) + *V* *a* *r*(log*O* *P*_*C*_).

## Appendix B Relationships between parameters of the ‐dimensional model

### B.1

In this section, we derive the relationships between the model hyper‐parameters (conditional variances $\psi_{1}^{2},\psi_{2}^{2},\ldots ,\psi_{N}^{2}$ and slopes $\lambda_{21},\lambda_{31},\lambda_{32},\ldots ,\lambda_{N1},\lambda_{N2},\ldots ,\lambda_{N(N-1)}$) and the between‐study parameters (correlations and standard deviations).

#### B.1 Model with unstructured covariance

In the model with the unstructured covariance the between‐study variances have the following forms:

(B.1) $$\left\{\begin{array}{l} \mathrm{Var}(\mu_{1})=\tau_{1}^{2}=\psi_{1}^{2}\\ \mathrm{Var}(\mu_{2})=\tau_{2}^{2}=\psi_{2}^{2}+\lambda_{21}^{2}\psi_{1}^{2}=\psi_{2}^{2}+\lambda_{21}^{2}\tau_{1}^{2}\\ \mathrm{Var}(\mu_{3})=\tau_{3}^{2}=\psi_{3}^{2}+\lambda_{31}^{2}\psi_{1}^{2}+\lambda_{32}^{2}(\psi_{2}^{2}+\lambda_{21}^{2}\psi_{1}^{2})=\psi_{3}^{2}+\lambda_{31}^{2}\tau_{1}^{2}+\lambda_{32}^{2}\tau_{2}^{2}\\ \vdots \\ \mathrm{Var}(\mu_{N})=\tau_{N}^{2}=\psi_{N}^{2}+\lambda_{N1}^{2}\tau_{1}^{2}+\cdots +\lambda_{N(N-1)}^{2}\tau_{N-1}^{2}, \end{array}\right.$$

the covariances are

(B.2) $$\left\{\begin{array}{l} \mathrm{Cov}(\mu_{1},\mu_{2})=\lambda_{21}\mathrm{Var}(\mu_{1})\\ \mathrm{Cov}(\mu_{1},\mu_{3})=\lambda_{31}\mathrm{Var}(\mu_{1})+\lambda_{32}\mathrm{Cov}(\mu_{2},\mu_{1})\\ \mathrm{Cov}(\mu_{2},\mu_{3})=\lambda_{32}\mathrm{Var}(\mu_{2})+\lambda_{31}\mathrm{Cov}(\mu_{2},\mu_{1})\\ \vdots \\ \mathrm{Cov}(\mu_{Q},\mu_{N})=\lambda_{\mathrm{NQ}}\mathrm{Var}(\mu_{Q})+\overset{N-1}{\underset{P=1,P\neq Q}{\sum }}\lambda_{\mathrm{NP}}\mathrm{Cov}(\mu_{P},\mu_{Q}),\;\;Q=1,\ldots ,N-1, \end{array}\right.$$

and corresponding correlations

(B.3) $$\left\{\begin{array}{l} \rho_{b}^{12}=\frac{\lambda_{21}\tau_{1}^{2}}{\sqrt{\tau_{1}^{2}\tau_{2}^{2}}}\\ \rho_{b}^{13}=\frac{\lambda_{31}\tau_{1}^{2}+\lambda_{32}\lambda_{21}\tau_{1}^{2}}{\sqrt{\tau_{1}^{2}\tau_{3}^{2}}}\\ \rho_{b}^{23}=\frac{\lambda_{32}\tau_{2}^{2}+\lambda_{31}\lambda_{21}\tau_{1}^{2}}{\sqrt{\tau_{2}^{2}\tau_{3}^{2}}}\\ \vdots \\ \rho_{b}^{\mathrm{QN}}=\frac{\lambda_{\mathrm{NQ}}\tau_{Q}^{2}+\overset{N-1}{\underset{P=1,P\neq Q}{\sum }}\lambda_{\mathrm{NQ}}\mathrm{Cov}(\mu_{P},\mu_{Q})}{\sqrt{\tau_{Q}^{2}\tau_{N}^{2}}},\;\;Q=1,\ldots ,N-1. \end{array}\right.$$

To obtain implied prior distribution for the hyper‐parameters, we need to express them in terms of the between‐study correlations and standard deviation. The conditional variances are obtained by rearranging terms in Equation (B.1) which gives formulae in (21). To obtain formulae for the slopes, the set of simultaneous Equations (B.3) needs to be solved giving the formulae in (22).

#### B.1 Model with structured covariance

In the model with structured covariance matrix, the correlations have a simple form,

(B.4) $$\left\{\begin{array}{l} \rho_{b}^{12}=\lambda_{21}\frac{\tau_{1}}{\tau_{2}}\\ \rho_{b}^{23}=\lambda_{32}\frac{\tau_{2}}{\tau_{3}}\\ \vdots \\ \rho_{b}^{(N-1)N}=\lambda_{N(N-1)}\frac{\tau_{\left(N-1\right)}}{\tau_{N}}, \end{array}\right.$$

which lead to also simpler (and easier to calculate and implement) relationships (25), between the slopes and the between‐study parameters.
