# Supplementary material for: Bayesian meta‐analytical methods to incorporate multiple surrogate endpoints in drug development process
Source: Stat Med. 2015 Nov 3;35(7):1063–89. doi: 10.1002/sim.6776 (PMC4950070; doi:10.1002/sim.6776)
Supplement: Supplementary file 1 — supporting Info Item [file SIM-35-1063-s001.pdf]

Research Article

Statistics in Medicine

Received XXXX

(www.interscience.wiley.com) DOI: 10.1002/sim.0000

Supporting Web Materials to Bayesian meta-analytical methods to incorporate multiple surrogate endpoints in drug development process

Sylwia Bujkiewicz[a]* John R.Thompson[b], Richard D Riley[c] and Keith R. Abrams[a]

A. WinBUGS and R programmes

A.1. WinBUGS code for trivariate meta-analysis with unstructured covariance

```
Model {
var[index,3]~dunif(0.0001,1000)
for (i in 1:num) {
Prec_w[i,1:3,1:3] <- inverse(delta[i,1:3,1:3])
#covariance matrix for the j-th study
delta[i,1,1]<-var[i,1]/n[i,1]
delta[i,2,2]<-var[i,2]/n[i,2]
delta[i,3,3]<-var[i,3]/n[i,3]
delta[i,1,2]<-sqrt(delta[i,1,1])*sqrt(delta[i,2,2])*rho_w_RM[i]
delta[i,2,1]<-delta[i,1,2]
delta[i,1,3]<-sqrt(delta[i,1,1])*sqrt(delta[i,3,3])*rho_w_DM[i]
delta[i,3,1]<-delta[i,1,3]
delta[i,2,3]<-sqrt(delta[i,2,2])*sqrt(delta[i,3,3])*rho_w_RD[i]
delta[i,3,2]<-delta[i,2,3]
}
# Random effects model
for (i in 1:num) {
Y[i,1:3]~dmnorm(mu[i,1:3], Prec_w[i,1:3,1:3])
# product normal formulation for the between study part:
mu[i,1]~dnorm(etaM,precM)
mu[i,2]~dnorm(etaR[i],precR)
etaR[i]<-lambdaR0+lambdaR1*mu[i,1]
mu[i,3]~dnorm(etaD[i],precD)
etaD[i]<-lambdaD0+lambdaD1*mu[i,1] +lambdaD2*mu[i,2]
```

[a]Biostatistics Research Group, Department of Health Sciences, University of Leicester, University Road, Leicester, LE1 7RH, UK
[b]Genetic Epidemiology Group, Department of Health Sciences, University of Leicester, University Road, Leicester, LE1 7RH, UK
[c]Research Institute of Primary Care and Health Sciences, Keele University, Staffordshire, ST5 5BG, UK
*Correspondence to: Department of Health Sciences, University of Leicester, University Road, Leicester, LE1 7RH, UK. E-mail: sb309@le.ac.uk

 Copyright © 0000 John Wiley & Sons, Ltd.

```
}
etaM~dnorm(0.0, 0.001)
lambdaD0~dnorm(0.0, 1.0E-3)
lambdaR0~dnorm(0.0, 1.0E-3)
corr.dis.mri~dunif(-0.99,0.99)
corr.rel.mri~dunif(-0.99,0.99)
corr.dis.rel~dunif(-0.99,0.99)
sd.mri~dnorm(0,0.1)I(0,)
sd.dis~dnorm(0,0.1)I(0,)
sd.rel~dnorm(0,0.1)I(0,)
gammaM.sq<-pow(sd.mri,2)
precM<-1/gammaM.sq
gammaR.sq<-pow(sd.rel,2) - pow(lambdaR1,2)*pow(sd.mri,2)
precR<-1/gammaR.sq
gammaD.sq<-pow(sd.dis,2) - pow(lambdaD1,2)*pow(sd.mri,2)
   - pow(lambdaD2,2)*pow(sd.rel,2)
precD<-1/gammaD.sq
mean.mri<-etaM
mean.rel<-lambdaR0+lambdaR1*etaM
mean.dis<-lambdaD0+lambdaD1*etaM+lambdaD2*mean.rel
lambdaR1<-corr.rel.mri*sd.rel/sd.mri
lambdaD1<-corr.dis.mri*sd.dis/sd.mri - lambdaD2*lambdaR1
lambdaD2<-(corr.dis.rel*sd.dis/sd.rel -
  corr.dis.mri*lambdaR1*sd.mri*sd.dis/pow(sd.rel,2))/
  (1-pow(lambdaR1,2)*pow(sd.mri,2)/pow(sd.rel,2))
arr<-exp(mean.rel)
mriirr<-exp(mean.mri)
disrr<-exp(mean.dis)
newTru<-mu[index,3]
}
```

A.2. WinBUGS code for trivariate meta-analysis with structured covariance

```
Model {
var[index,3]~dunif(0.0001,1000)
for (i in 1:num) {
Prec_w[i,1:3,1:3] <- inverse(delta[i,1:3,1:3])
#covariance matrix for the j-th study
delta[i,1,1]<-var[i,1]/n[i,1]
delta[i,2,2]<-var[i,2]/n[i,2]
delta[i,3,3]<-var[i,3]/n[i,3]
delta[i,1,2]<-sqrt(delta[i,1,1])*sqrt(delta[i,2,2])*rho_w_RM[i]
delta[i,2,1]<-delta[i,1,2]
delta[i,1,3]<-sqrt(delta[i,1,1])*sqrt(delta[i,3,3])*rho_w_DM[i]
delta[i,3,1]<-delta[i,1,3]
delta[i,2,3]<-sqrt(delta[i,2,2])*sqrt(delta[i,3,3])*rho_w_RD[i]
delta[i,3,2]<-delta[i,2,3]
}
# Random effects model
for (i in 1:num) {
Y[i,1:3]~dmnorm(mu[i,1:3], Prec_w[i,1:3,1:3])
# product normal formulation for the between study part:
mu[i,1]~dnorm(etaM,precM)
mu[i,2]~dnorm(etaR[i],precR)
etaR[i]<-lambdaR0+lambdaR1*mu[i,1]
mu[i,3]~dnorm(etaD[i],precD)
```

 Copyright © 0000 John Wiley & Sons, Ltd.

```
etaD[i]<-lambdaD0+lambdaD2*mu[i,2]
}
etaM~dnorm(0.0, 0.001)
lambdaD0~dnorm(0.0, 1.0E-3)
lambdaR0~dnorm(0.0, 1.0E-3)
corr.dis.mri~dunif(-0.99,0.99)
corr.rel.mri~dunif(-0.99,0.99)
corr.dis.rel~dunif(-0.99,0.99)
sd.mri~dnorm(0,0.1)I(0,)
sd.dis~dnorm(0,0.1)I(0,)
sd.rel~dnorm(0,0.1)I(0,)
gammaM.sq<-pow(sd.mri,2)
precM<-1/gammaM.sq
gammaR.sq<-pow(sd.rel,2) - pow(lambdaR1,2)*pow(sd.mri,2)
precR<-1/gammaR.sq
gammaD.sq<-pow(sd.dis,2) - pow(lambdaD1,2)*pow(sd.mri,2)
   - pow(lambdaD2,2)*pow(sd.rel,2)
precD<-1/gammaD.sq
mean.mri<-etaM
mean.rel<-lambdaR0+lambdaR1*etaM
mean.dis<-lambdaD0+lambdaD2*mean.rel
lambdaR1<-corr.rel.mri*sd.rel/sd.mri
lambdaD2<-corr.dis.rel*sd.dis/sd.rel
arr<-exp(mean.rel)
mriirr<-exp(mean.mri)
disrr<-exp(mean.dis)
newTru<-mu[index,3]
}
```

This model reduces easily to the bivariate meta-analysis.

Copyright © 0000 John Wiley & Sons, Ltd.

B. Additional tables

Table 1. Results of validation obtained from the model with unstructured between-study covariance matrix.

omitted study for validation	observed	λ_{20}	λ_{21}	ψ_2^2	λ_{30}	λ_{31}	λ_{32}	ψ_3^2	predicted mean (95%CrI)
Paty (A)	1.00	-0.28	0.26	0.15	-0.10	0.01	0.41	0.03	0.86 (0.43, 1.73)
Paty (B)	0.64	-0.28	0.26	0.15	-0.07	0.00	0.45	0.02	0.77 (0.38, 1.54)
Simon	0.53	-0.28	0.26	0.15	-0.04	0.05	0.40	0.02	0.80 (0.36, 1.77)
Millefiorini	0.13	-0.28	0.26	0.15	-0.07	0.04	0.36	0.02	0.65 (0.12, 3.62)
Li (C)	0.73	-0.28	0.26	0.15	-0.08	0.00	0.46	0.03	0.79 (0.45, 1.41)
Li (D)	0.63	-0.28	0.27	0.15	-0.08	-0.05	0.53	0.02	0.81 (0.44, 1.50)
Polman	0.50	-0.27	0.27	0.15	-0.12	0.00	0.33	0.02	0.61 (0.35, 1.07)
Comi (E)	0.64	-0.28	0.26	0.15	-0.09	-0.01	0.47	0.03	0.62 (0.36, 1.08)
Comi (F)	0.69	-0.28	0.26	0.15	-0.08	0.00	0.46	0.03	0.64 (0.38, 1.09)
Rudick	0.73	-0.28	0.26	0.15	-0.07	0.04	0.44	0.03	0.61 (0.37, 1.01)
Sorensen	0.57	-0.28	0.26	0.15	-0.08	0.01	0.43	0.02	0.64 (0.23, 1.82)
Clanet	1.00	-0.27	0.27	0.15	-0.15	-0.02	0.40	0.03	0.88 (0.49, 1.58)
Mikol	1.39	-0.27	0.27	0.15	-0.19	-0.01	0.32	0.02	0.83 (0.45, 1.53)

Table 2. Results of validation obtained from the model with structured between-study covariance matrix.

study	observed	λ_{20}	λ_{21}	ψ_2^2	λ_{30}	λ_{31}	ψ_3^2	predicted mean (95%CrI)
Paty (A)	1.00	-0.21	0.33	0.14	-0.09	0.46	0.03	0.88 (0.44, 1.73)
Paty (B)	0.64	-0.22	0.32	0.14	-0.05	0.49	0.02	0.77 (0.38, 1.54)
Simon	0.53	-0.21	0.33	0.14	-0.05	0.48	0.02	0.79 (0.37, 1.72)
Millefiorini	0.13	-0.21	0.33	0.14	-0.06	0.46	0.02	0.62 (0.11, 3.44)
Li (C)	0.73	-0.21	0.33	0.15	-0.05	0.49	0.03	0.80 (0.45, 1.42)
Li (D)	0.63	-0.22	0.32	0.14	-0.04	0.50	0.02	0.79 (0.45, 1.39)
Polman	0.50	-0.21	0.33	0.14	-0.09	0.41	0.02	0.58 (0.33, 1.03)
Comi (E)	0.64	-0.22	0.32	0.14	-0.06	0.49	0.03	0.62 (0.36, 1.07)
Comi (F)	0.69	-0.21	0.33	0.14	-0.07	0.50	0.03	0.63 (0.37, 1.08)
Rudick	0.73	-0.22	0.32	0.14	-0.07	0.53	0.03	0.61 (0.38, 0.98)
Sorensen	0.57	-0.21	0.32	0.14	-0.06	0.48	0.02	0.63 (0.24, 1.66)
Clanet	1.00	-0.22	0.32	0.14	-0.10	0.44	0.03	0.92 (0.53, 1.60)
Mikol	1.39	-0.22	0.32	0.15	-0.16	0.36	0.02	0.85 (0.46, 1.57)

Copyright © 0000 John Wiley & Sons, Ltd.

C. R code for the simulation

C.1. Simulation from the Normal distribution

```
rho_b12<-0.8
rho_b13<-0.8
rho_b23<-0.8
tau1<-0.5
tau2<-0.5
tau3<-0.5
# coefficients for TRMA UCM:
lam21<-tau2*rho_b12/tau1
lam32<-(rho_b23*tau2*tau3-rho_b13*tau3*tau1*lam21)/(tau2**2-lam21**2 *
tau1**2)
lam31<-tau3*rho_b13/tau1-lam32*lam21
# alternatively for TRMA SCM
#lam32<-rho_b23*tau3/tau2
psi1<-tau1
psi2<-sqrt(tau2**2 - lam21**2 * tau1**2)
psi3<-sqrt(tau3**2 - lam31**2 * tau1**2 - lam32**2 * tau2**2)
# alternatively for TRMA SCM
# psi3<-sqrt(tau3**2 - lam32**2 * tau2**2)
eta1<- -0.3
mpmri<-30
vpmri<-420
mprel<-150
vprel<-15000
mpdis<-25
vpdis<-275
alpha1<-mpmri*mpmri/vpmri
alpha2<-mprel*mprel/vprel
alpha3<-mpdis*mpdis/vpdis
xi1<-mpmri/vpmri
xi2<-mprel/vprel
xi3<-mpdis/vpdis
sc1<-1/xi1
sc2<-1/xi2
sc3<-1/xi3
lam20<--0.3
lam30<--0.1
num<-15
rho12<-rho13<-rho23<-array(0,num)
rho12<-c(rep(0.275,num))
rho13<-c(rep(0.1,num))
rho23<-c(rep(0.1,num))
m1<-m2<-m3<-s1<-s2<-s3<-var1<-var2<-var3<-prec1<-prec2<-prec3<-array(0,num)
sigma <- array(matrix(0,3,3),num)
y<-matrix(,num,3)
for (i in 1:num){
  ll<-5
  while (ll>0) {
  m1[i]<-rnorm(1,eta1,psi1)
  m2[i]<-rnorm(1,lam20+lam21*m1[i],psi2)
  m3[i]<-rnorm(1,lam30+lam31*m1[i]+lam32*m2[i],psi3)
  # alternatively for TRMA SCM:
  # m3[i]<-rnorm(1,lam30+lam32*m2[i],psi3)
  prec1[i]<-rgamma(1,shape=alpha1,scale = sc1)
```


Copyright © 0000 John Wiley & Sons, Ltd.

```
  prec2[i]<-rgamma(1,shape=alpha2,scale = sc2)
  prec3[i]<-rgamma(1,shape=alpha3,scale = sc3)
  var1[i]<-1/prec1[i]
  var2[i]<-1/prec2[i]
  var3[i]<-1/prec3[i]
  s1[i]<-sqrt(var1[i])
  s2[i]<-sqrt(var2[i])
  s3[i]<-sqrt(var3[i])
  sigma<- matrix(c(var1[i],s1[i]*s2[i]*rho12[i],s1[i]*s3[i]*rho13[i],
    s2[i]*s1[i]*rho12[i], var2[i], s2[i]*s3[i]*rho23[i],
    s3[i]*s1[i]*rho13[i], s3[i]*s2[i]*rho23[i], var3[i]),3,3)
  y[i,]<-mvrnorm(n=1, c(m1[i],m2[i],m3[i]), sigma)
  l1<-(s1[i]>2.0)
  l2<-(s2[i]>2.0)
  l3<-(s3[i]>2.0)
  ll<-l1+l2+l3
  }
}
```

C.2. Simulation from the t-distribution

The following substitution need to me made in the above R code for the simulations when simulating from the *t*-distribution.

```
df<-4
...
while (ll>0) {
m1[i]<-rt(1,df=df)*psi1*sqrt((df-2)/df) + eta1
tmp1<-lam20+lam21*m1[i]
m2[i]<-rt(1,df=df)*psi2*sqrt((df-2)/df) + tmp1
tmp2<-lam30+lam31*m1[i]+lam32*m2[i]
m3[i]<-rt(1,df=df)*psi3*sqrt((df-2)/df) + tmp2
# alternatively for TRMA SCM:
#tmp2<-lam30+lam32*m2[i]
#m3[i]<-rt(1,df=df)*psi3*sqrt((df-2)/df) + tmp2
...
sigma<- matrix(c(var1[i],s1[i]*s2[i]*rho12[i],s1[i]*s3[i]*rho13[i],
  s2[i]*s1[i]*rho12[i], var2[i], s2[i]*s3[i]*rho23[i],
  s3[i]*s1[i]*rho13[i], s3[i]*s2[i]*rho23[i], var3[i]),3,3)*(df-2)/df
y[i,]<-rmvt(n=1, delta=c(m1[i],m2[i],m3[i]), sigma=sigma, df=df)
...
}
```

C.3. Simulation from the mixture of Normal distributions

The following addition to the code in Section C.1 must be made when simulating from the mixture of Normal distributions.

```
u<-runif(num)
for (i in 1:num){
  ll<-5
  while (ll>0) {
  if (u[i]<0.3){eta1<-eta-4*psi1
    } else if (u[i]<0.8){
      eta1<-eta
    }else{
      eta1<-eta+4*psi1
    }
  ...
  }
}
```

 Copyright © 0000 John Wiley & Sons, Ltd.

D. Additional graphs for scenarios with four surrogates

Figure 1. Scenarios of modelling multiple surrogate endpoints with varying correlation structure.

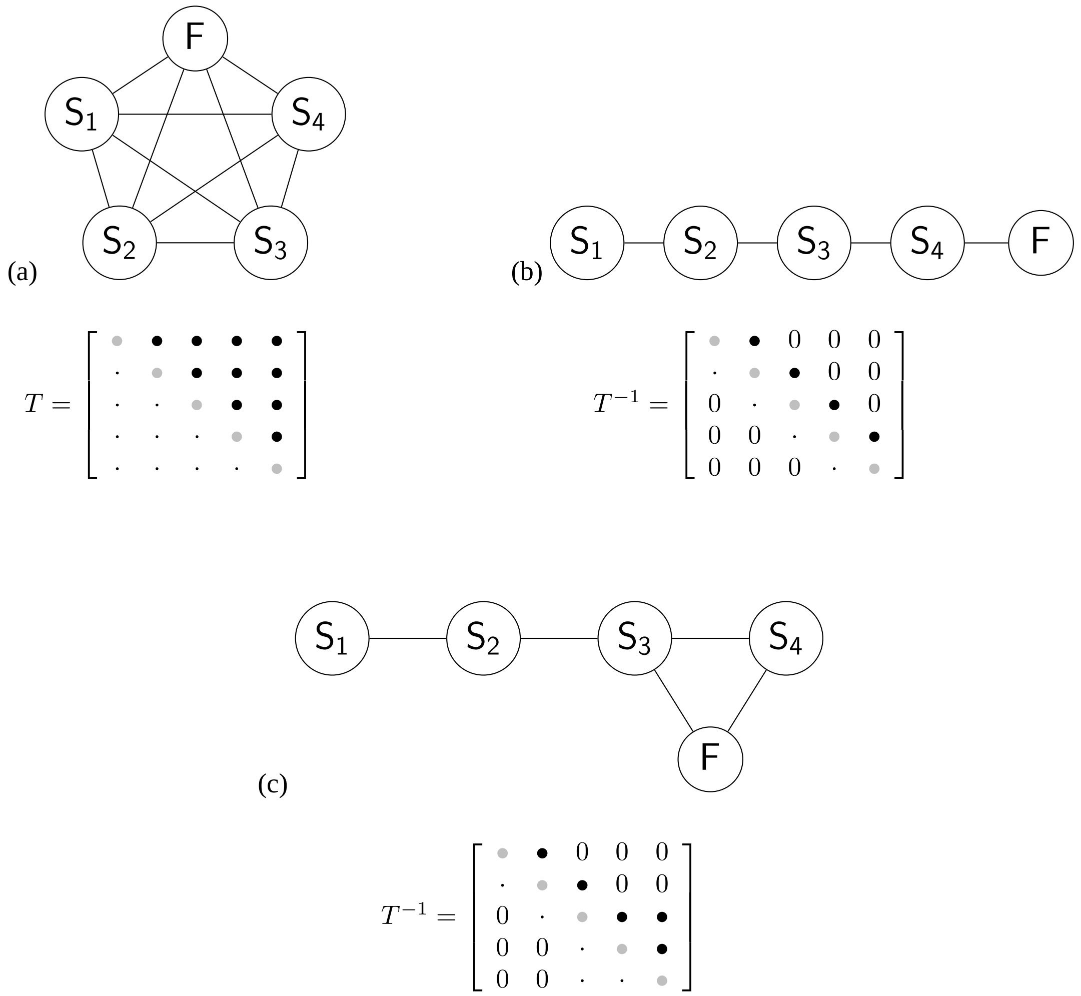

Copyright © 0000 John Wiley & Sons, Ltd.
